# CAR T cell-driven induction of iNOS in tumor-associated macrophages promotes CAR T cell resistance in B cell lymphoma

**DOI:** 10.21203/rs.3.rs-3481746/v1

**Published:** 2025-03-31

**Authors:** Marco Davila, Sae Bom Lee, Yun Pyo Kang, Justin Boucher, Jay Mandula, Emiliano Roselli, Darwin Chang, Rachel Jimenez, Hiroshi Kotani, Kayla Reid, Julio Vazquez-Martinez, Nolan Beatty, Payal Goala, Rosa Sierra-Mondragon, Min Liu, John Koomen, Jonathan Nguyen, Mohammad Hussaini, Timothy Shaw, Xuefeng Wang, Rawan Faramand, Michael Jain, Frederick Locke, Paulo Rodriguez, Cooper Sailer, Shannon McSain, Showkat Hamid, Muhammad Tariq, Jianmin Wang, Julieta Abraham-Miranda

## Abstract

Chimeric antigen receptor (CAR) T cell therapies have revolutionized B cell malignancy treatment, but subsets of patients with large B cell lymphoma (LBCL) experience primary resistance or relapse after CAR T cell treatment. To uncover tumor microenvironment (TME)-induced resistance mechanisms, we examined patients’ intratumoral immune infiltrates and observed that elevated levels of immunoregulatory macrophages in pre-infusion tumor biopsies are correlated with poor clinical responses. CAR T cell-produced interferon-gamma (IFN-γ) promotes the expression of inducible nitric oxide synthase (iNOS, NOS2) in immunoregulatory macrophages, impairing CAR T cell function. Mechanistically, iNOS-expressing macrophages upregulated the p53 pathway, mediating apoptosis and cell cycle arrest in CAR T cells, while downregulating the MYC pathway involved in ribosome biogenesis and protein synthesis. Furthermore, CAR T cell metabolism is compromised by depletion of glycolytic intermediates and rewiring of the TCA cycle. Pharmacological inhibition of iNOS enhances the CAR T cell treatment efficacy in B cell tumor-bearing mice. Notably, elevated levels of iNOS+CD14+ monocytes were observed in leukaphereses of patients with non-durable response to CAR T cell therapy. These findings suggest that mitigating iNOS in tumor-associated macrophages (TAMs) by blocking IFN-γ secretion from CAR T cells will improve outcomes for LBCL patients.

## INTRODUCTION

The success of CD19-targeted CAR T cell therapies has advanced the treatment of B cell malignancies [1-7]. However, a substantial proportion of patients with LBCL experience primary resistance or relapse, supporting the need to augment CAR T cell efficacy [8-11]. Factors hindering the effectiveness of CAR T cell therapy include a high tumor burden prior to CAR T cell infusion [12,13], loss of or decreased CD19 expression on tumor cells [14,15], tumor genetic alterations [16,17], and the highly differentiated or dysfunctional state of CAR T cells [18,19]. Recent studies have also emphasized the importance of the TME in determining clinical outcomes in CAR T therapy patients [20,21].

The TME of B cell lymphoma contains various immune cell types [22,23], including macrophages, myeloid-derived suppressor cells (MDSCs), and regulatory T cells (Tregs), which can impede the recruitment, expansion, and activity of T cells, including endogenous T cells and infused CAR T cells. In LBCL, the pre-infusion TME, characterized by elevated expression of genes associated with immune suppression and diminished T cell-related signatures, is correlated with relapse after CAR T cell therapy [21]. In contrast, higher rates of complete response are associated with a TME that exhibits immune gene signatures linked to cytotoxic T cell activation and is enriched with chemokines and cytokines that potentiate T cell involvement.

Macrophages perform diverse biological functions in response to tissue pathophysiology and environmental cues, acting as central mediators in immune responses [24]. They are key contributors to immune-mediated toxicities associated with CAR T cell therapy, such as the cytokine release syndrome

(CRS), which is in part mediated through the release of inflammatory cytokines by myeloid cells [25-27]. Activated macrophages secrete proinflammatory cytokines TNF-α, IL-6, and IL-1β and upregulate inducible nitric oxide synthase (iNOS) and nitric oxide (NO), exacerbating CRS through induction of endothelial dysfunction and vascular leakage [26]. Additionally, macrophages facilitate cancer progression in various cancers by suppressing T cell effector function through multiple processes, including expression of inhibitory checkpoint ligands such as programmed cell death-ligand 1 (PD-L1), secretion of inhibitory cytokines such as TGF-b and IL-10, and depletion of amino acids, including arginine and tryptophan [28-31]. However, the crosstalk between CAR T cells and macrophages as a mechanism of therapeutic resistance remains poorly defined.

Here, we report the reciprocal interactions between CAR T cells and macrophages, contributing to CAR T cell therapy resistance in B cell lymphoma. Our findings demonstrate that IFN-g produced by CAR T cells induces phenotypic changes in macrophages, amplifying their immunoregulatory potential. Notably, iNOS induction in macrophages drives CAR T cell dysfunction by impairing their functional and metabolic capacities. This study uncovers a critical counter-regulatory mechanism in which IFN-g-producing CAR T cells restricts therapeutic efficacy. These insights highlight the targetable macrophage-driven resistance mechanisms to improve CAR T cell therapy outcomes in B cell lymphoma.

## RESULTS

#### Macrophages in the pre-CAR T cell treatment TME are linked to therapeutic responses in LBCL patients

We examined the tumor immune infiltrate and its relationship with clinical outcomes in patients with LBCL receiving Axicabtagene ciloleucel (axi-cel). Bulk RNA sequencing (RNA-seq) was performed on patient tumor biopsies taken before lymphodepletion and CAR T cell treatment. Subsequently, CIBERSORTx was used to deconvolute intratumoral immune cell composition (**Fig. 1A**) [32,33]. We found that patients with non-durable responses (NDR) to CAR T cell therapy, characterized by lymphoma relapse or death from disease, exhibited a higher proportion of transcriptionally identified M2-like macrophages compared to patients with durable responses (DR), who remained in remission for at least 6 months following axi-cel infusion (**Fig. 1B**). Similarly, gene set enrichment analysis (GSEA) revealed the enrichment of M2 macrophage-associated genes in patients with NDR (**Fig. 1C**). The proportion of nonactivated macrophages (M0) was lower in patients with NDR, while levels of M1-like macrophages were similar between patients with NDR and DR (**Fig. 1B**). Furthermore, we observed that a higher abundance of M2-like macrophages in patients correlated with worse progression-free survival after axi-cel therapy (**Fig. 1D**). These findings collectively indicate that the presence of M2-like macrophages within the TME prior to CAR T cell therapy is associated with poor therapeutic responses to axi-cel in patients with LBCL.

#### Immunoregulatory actions of macrophages on CAR T cells

To explore how macrophages may impact the cellular function of CAR T cells, we employed a syngeneic coculture system (**Fig. 2A**). In this model, murine anti-CD19 CAR T cells [34], which include CD28 and CD3z signaling domains linked to a fluorescent mCherry reporter (1928z) (**Extended Data Fig. 1A**), were cocultured with a murine malignant B cell line (Em-myc cells) in the presence or absence of mouse bone marrow-derived macrophages (BMDMs). The BMDMs were either unpolarized (unMac) or activated with type 2 cytokines IL-4 and IL-10 to exhibit an M2-like phenotype. We have termed these activated macrophages as 'imMac' to emphasize their distinctive immunoregulatory activity when cocultured with CAR T cells. CAR T cells cocultured with imMac showed increased cell death (**Fig. 2B**) and reduced DNA replication (**Fig. 2C**) compared to CAR T cells cocultured with unMac or without macrophages. Correspondingly, CAR T cells exhibited diminished expansion during coculture with imMac (**Fig. 2D** and **Extended Data Fig. 1B**). Moreover, CAR T cells cocultured with imMac showed lower total CAR expression (**Fig. 2E**) as well as reduced surface CAR expression (**Fig. 2F**). We next explored the impact of imMac on CAR T cell effector function. To exclude the direct contribution of macrophage effector activities in these functional assays, we first cocultured CAR T cells, Em-myc cells, and macrophages for 48 hours (**Fig. 2G**). Next, CAR T cells isolated from the cocultures were evaluated for their effector function against fresh Em-myc cells. CAR T cells derived from cocultures with imMac exhibited impaired production of effector cytokines IFN-g and tumor necrosis factor-alpha (TNF-a) (**Fig. 2H** and **I**) and demonstrated decreased ability to lyse target tumor cells (**Fig. 2J**). Collectively, these results show that macrophages polarized towards an M2-like phenotype exert immunoregulatory actions that impair multiple aspects of CAR T cell biology, including survival, expansion, and CAR-dependent effector functions.

### CAR T cell-exposed imMac upregulates iNOS

We next interrogated the metabolic crosstalk between CAR T cells and imMac to investigate the potential involvement of immune-metabolic alterations. We conducted a comprehensive analysis of metabolite profiles in the supernatants collected from our coculture model via global metabolomics using liquid chromatography-mass spectrometry (LC-MS) (**Fig. 3A**). We observed a significant increase in citrulline and ornithine and a concomitant reduction in arginine within the supernatants derived from cocultures containing imMac compared to cocultures containing unMac or no macrophages (No Mac) (**Fig. 3B-F**). Macrophages possess the capacity to metabolize arginine through arginase-1 (ARG-1) or iNOS, producing ornithine and urea or citrulline and NO, respectively [35] (**Extended Data Fig. 2A**). We observed that in the absence of CAR T cells, imMac exhibited high expression levels of ARG-1 but minimal iNOS, whereas unMac displayed minimal expression of both ARG-1 and iNOS (**Fig. 3G**). When CAR T cells were cocultured with macrophages, there was a significant induction of iNOS in imMac and, to a lesser extent, in unMac. The expression levels of ARG-1 in unMac and imMac remained unchanged, regardless of the presence of CAR T cells in the cocultures. Neither $CD3^+$ T cells nor Em-myc cells expressed ARG-1 or iNOS, confirming that the expression of these enzymes was limited to macrophages in this model (**Extended Data Fig. 2B**). Consistent with the enhanced iNOS in imMac, higher levels of NO were produced in cocultures with imMac compared to cocultures with unMac or without macrophages

(**Fig. 3H**). Additionally, coculture with CAR T cells substantially increased the expression of PD-L1 in both unMac and imMac (**Extended Data Fig. 2C**). Together, these findings demonstrate that exposure of imMac to CAR T cells induces phenotypic changes in imMac, including enhanced arginine metabolism through the upregulation of iNOS.

#### iNOS upregulation in imMac drives suppression of CAR T cell function

To investigate whether arginine metabolism by imMac contributes to the impairment of CAR T cell function, we examined the effects of ARG-1 and iNOS inhibitors (**Extended Data Fig. 3A**). Treatment with the ARG-1 inhibitor nor-NOHA [36] did not restore CAR T cell expansion in cocultures with imMac (**Extended Data Fig. 3B**). The supraphysiological arginine concentration (1.15 mM) in culture media could diminish arginine depletion effect by ARG-1, thus we repeated the coculture at the physiological arginine concentration (150 μM) [37]. However, even in the context of physiological arginine levels, treatment with nor-NOHA failed to rescue CAR T cell expansion (**Extended Data Fig. 3C**), suggesting ARG-1 by itself is not sufficient to inhibit CAR T cells. Next, we treated the cocultures with the iNOS inhibitor L-NIL [25,38] and observed rescue of CAR T cell expansion in cocultures with imMac (**Fig. 4A**). Furthermore, L-NIL treatment preserved the capacity of CAR T cells to kill tumor cells (**Fig. 4B**) and produce the effector cytokines IFN-g and TNF-a (**Fig. 4C** and **D**). Moreover, imMac developed from iNOS-deficient (iNOS$^{-/-}$) mice BMDMs did not inhibit CAR T cell expansion (**Fig. 4E**) or impair CAR T cell tumor killing capacity (**Fig. 4F**). Importantly, iNOS$^{-/-}$ imMac expressed similar levels of ARG-1 and PD-L1 as wild-type (WT) imMac (**Extended Data Fig. 3D**), indicating that these factors were not responsible for the suppression of CAR T cell function by imMac. Inhibition or genetic ablation of iNOS attenuated the production of NO (**Extended Data Fig. 3E** and **F**) and citrulline (**Extended Data Fig. 3G)** in the cocultures with imMac. The levels of arginine and ornithine remained comparable regardless of iNOS ablation or inhibition (**Extended Data Fig. 3H** and **I**), ruling out their altered levels as the drivers of CAR T cell impairment. These findings collectively demonstrate that imMac suppresses CAR T cell function through the enzymatic activity of iNOS.

We further investigated whether the iNOS products, citrulline and NO, were responsible for CAR T cell suppression by imMac. Exposure to high levels of citrulline did not impact the expansion of CAR T cells (**Extended Data Fig. 3J**). NO reacts with superoxide to form peroxynitrite (PNT, $ONOO^-$), which leads to protein oxidation, lipid peroxidation, and DNA damage [39]. Treatment with the NO-donor NCX-4016 or PNT resulted in the inhibition of CAR T cell expansion (**Fig. 4G** and **H**) and diminished ability of CAR T cells to kill tumor cells (**Fig. 4I**) and secrete effector cytokines IFN-g and TNF-a (**Extended Data Fig. 3K** and **L**). Notably, treatment with NO-scavenger carboxyl-PTIO (c-PTIO) partially rescued the expansion of CAR T cells during cocultures with imMac (**Fig. 4J**). These data indicate that NO and PNT act as key mediators of iNOS-induced dysfunction of CAR T cells.

#### CAR T cell-derived IFN-γ induces iNOS in imMac

CAR T cells secrete cytokines, such as IFN-γ and TNF-α, that activate macrophages [40,41]. A previous study reported that CAR T cell-derived IFN-γ upregulated iNOS in TAMs and their secretion of chemokines that enabled further recruitment of CAR T cells in a lung adenocarcinoma mouse model [42]. However, we found that neutralization of IFN-g with blocking antibodies attenuated CAR T cell-triggered iNOS expression in unMac and imMac (**Fig. 5A**) and reduced the production of NO (**Fig. 5B**). Furthermore, treatment of anti-IFN-g enhanced CAR T cell expansion in cocultures with imMac (**Fig. 5C**) and preserved the ability of CAR T cells to lyse tumor cells (**Fig. 5D**) and produce IFN-g and TNF-a (**Fig. 5E** and **F**). Consistently, CAR T cells deficient in IFN-g (IFN-$g^{-/-}$ CAR T cells) neither induced iNOS expression in unMac and imMac (**Extended Data Fig. 4A**) nor induced NO production in cocultures (**Extended Data Fig. 4B)**. Moreover, IFN-$g^{-/-}$ CAR T cells exhibited enhanced expansion during cocultures with imMac (**Extended Data Fig. 4C**). These findings demonstrate that blocking IFN-g production by CAR T cells mitigates the counter-regulatory iNOS-driven inhibitory effects of imMac.

To investigate the regulatory genes and pathways underlying the detrimental interactions between CAR T cells and imMac, we performed single-cell RNA sequencing (scRNA-seq) on cocultures of wild-type (WT) or *IFN-*$g^{-/-}$ CAR T cells with unMac or imMac (**Fig. 5G**). Unsupervised clustering analysis revealed distinct subpopulations of T cells and macrophages (**Extended data Fig. 5A-C**). Among the six major macrophage subpopulations identified, cluster 0 was the most predominant subset in imMac cocultured with WT CAR T cells (**Extended data Fig. 5D**). This cluster was characterized by elevated expression of Nos2/iNOS and inhibitory checkpoint ligands such as *Cd274/PD-L1* and *Pdcd1lg2/PD-L2* (**Extended data Fig. 5E**). Additionally, cluster 0 exhibited increased expression of immunosuppressive genes, including *Ptgs2/Cox-2*, *Entpd1/CD39*, *Il18bp, Fgl2*, and *Mertk* (**Extended data Fig. 5E**). Pathway analysis of genes upregulated in imMac cocultured with WT CAR T cells revealed significant enrichment of the IFN-g and IFN-a response pathways, as well as the hypoxia-inducible factor (HIF)-1a pathway (**Extended data Fig. 5F**). Notably, increased *Nos2/iNOS* expression within macrophage subclusters strongly correlated with HIF-1a pathway activation, suggesting HIF-1α signaling mediates *Nos2/iNOS* induction in imMac (**Extended data Fig. 5G and H**) [43,44]. These findings indicate that CAR T cell-derived IFN-γ reshapes the transcriptomic landscape of imMac, enhancing their immunoregulatory potential. Additionally, pathway analysis revealed enrichment of the HIF-1a and p53 pathways in T cells from WT CAR T cells cocultured with imMac, suggesting that imMac induces hypoxia-like conditions and cellular stress responses in CAR T cells, which likely also contributes to their functional impairment (**Fig. 5H)**.

#### iNOS-expressing imMac induces CAR T cell metabolic dysregulation

To elucidate the mechanisms underlying CAR T cell dysfunction induced by imMac, we performed proteomics analysis on CAR T cells from our culture model (**Fig. 6A**). Principal component analysis (PCA) revealed distinct protein profiles in CAR T cells cocultured with imMac compared to CAR T cells cocultured with unMac or without macrophages, with these differences reversed by L-NIL treatment (**Fig. 6B**). Consistent with the scRNA-seq data (**Fig. 5H**), pathway analysis demonstrated enrichment of the hypoxia and p53 pathways in CAR T cells cocultured with imMac (**Fig. 6C**). Notably, we observed an increase in p53 target proteins associated with cell cycle arrest (Cdkn1a/p21and Gtse1), apoptosis (Bbc3/PUMA, Apaf-1, Fas, Bax, Casp6, and Casp7), and DNA damage repair (Mgmt, Ercc5, Polk, and Xpc) (**Fig. 6D-G**). Phosphoproteomics analysis further revealed increased phosphorylation of Cdkn1a/p21 at Serine 78, which can enhance its ability to inhibit cyclin-dependent kinases (CDKs) and halt the cell cycle (**Extended Data Fig. 6A-D**) [45]. Additionally, pathway analysis identified downregulation of MYC target proteins in CAR T cells cocultured with imMac (**Fig. 6C**). Specifically, we observed reduced expression of MYC targets involved in ribosome biogenesis (Rsl1d1, Lsm2, Pwp1, Nolc1, Rpl18, and Rplp0), translation (Eif3j1/Eif3j2, Eif4a1, Hnrnpa2b1, Eef1b2, Etf1, Abce1, Rack1, Vbp1, and Hsp90ab1), and amino acid uptake (Slc7a5, Slc1a5, and Slc3a2) (**Fig. 6D-F** and **H**) [46]. Collectively, these findings support that iNOS-expressing imMac induces gene expression programs in CAR T cells that impair protein synthesis, cell cycle progression, and survival, ultimately dysregulating their antitumor efficacy.

Both the scRNA-seq and proteomics analyses identify cellular phenotypic changes associated with reduced cell cycle progression and survival. Cellular metabolism plays a crucial role in supporting rapid proliferation and effector function of T cells [47,48]. To investigate if metabolic dysregulation of CAR T cells is also induced by imMac, we conducted global metabolomics analysis on CAR T cells from our coculture model. We found significant alterations in glycolytic and TCA cycle intermediates in CAR T cells cocultured with imMac, which were restored with L-NIL treatment (**Fig. 7A-D**). Specifically, there was a marked depletion of glycolytic intermediates such as fructose 1,6-bisphosphate (F1,6BP), glyceraldehyde 3-phosphate (G3P), and dihydroxyacetone phosphate (DHAP) (**Fig. 7E-G**). Concurrently, there was an iNOS-dependent accumulation of TCA cycle metabolites citrate, aconitate, and succinate, along with a decrease in malate (**Fig. 7H-K**). Additionally, we observed a substantial accumulation of itaconate in CAR T cells cocultured with imMac (**Fig. 7L**). Itaconate is synthesized from aconitate via immune response gene 1 (IRG1), also known as aconitate decarboxylase (ACOD1), in tumor-associated myeloid cells and uptake of itaconate by $CD8^+$ T cells has been shown to suppress their proliferation and cytolytic activity [49]. Exposure of CAR T cells to a cell-permeant form of itaconate, 4-octyl itaconate (4-OI) [50], impaired their expansion (**Fig. 7M**). Given the concurrent accumulation of itaconate with citrate and aconitate in CAR T cells cocultured with imMac, we investigated whether CAR T cells can produce itaconate. Through $^{13}C_6$-glucose tracing on CAR T cells, we identified iNOS-dependent accumulation of $^{13}C$-labeled citrate, aconitate, and itaconate, as well as a reduction of $^{13}C_6$-labeled a-ketoglutarate (aKG), fumarate, and malate in CAR T cells cocultured with imMac (**Extended Data Fig. 7A-G**). Immunoblot analysis confirmed increased expression of IRG1 and decreased expression of isocitrate dehydrogenase 2 (IDH2) in CAR T cells cocultured with imMac (**Extended Data Fig. 7H**). These results indicate that imMac, via iNOS, depletes glycolytic intermediates and rewires the TCA cycle to divert aconitate towards itaconate production instead of aKG. These metabolic alterations were further corroborated by extracellular flux analysis, which revealed attenuated glycolytic and oxidative metabolic activities in CAR T cells cocultured with imMac, as evidenced by decreased extracellular acidification rate (ECAR) and oxygen consumption rate (OCR), which are proxies for glycolytic rate and mitochondrial oxidative phosphorylation, respectively (**Fig. 7N**). Importantly, L-NIL treatment preserved glycolytic and oxidative metabolic capacities, mitigating the metabolic disruptions caused by imMac.

#### iNOS inhibition improves CAR T cell therapeutic efficacy

We proceeded to investigate whether iNOS can limit the effectiveness of CAR T cell therapy in vivo. To eliminate the effects of tumor antigen spreading on bystander T cells and secondary sources of IFN-g production, we utilized *Rag1*$^{-/-}$ mice, which lack endogenous T and B cells. Em-myc B cell tumors were established in the peritoneal cavity (**Fig. 8A)**. CAR T cells carrying a truncated CD3z signaling domain (19dz) were used as non-functional controls (**Extended Data Fig. 2A**). Intraperitoneal transfer of WT 1928z, IFN-g$^{-/-}$ 1928z, or WT 19dz CAR T cells was performed to facilitate direct interaction with macrophages at the tumor site. We found that the frequency of iNOS$^{+}$ macrophages was significantly elevated in WT 1928z CAR T cell-treated mice compared to mice treated with IFN-g$^{-/-}$ 1928z or WT 19dz CAR T cells (**Fig. 8B**). The frequencies of ARG1$^{+}$ macrophages and total F4/80$^{+}$ CD11b$^{+}$ macrophages were similar across all groups (**Fig. 8C** and **D)**. Furthermore, CD11b$^{+}$ myeloid cells from the peritoneal cavity of WT 1928z CAR T cell-treated tumor-bearing mice suppressed expansion of fresh antigen-naïve CAR T cells *ex vivo* in an iNOS-dependent manner (**Fig. 8E**). We next assessed whether inhibition of iNOS could improve therapeutic efficacy of CAR T cells (**Fig. 8F**). Mice treated with a combination of 1928z CAR T cells and L-NIL exhibited significantly improved survival compared to mice treated with 1928z CAR T cells alone (**Fig. 8G**). Similarly, in C57BL/6 immune-competent mice bearing Em-myc B cell tumors, combinatorial treatment with 1928z CAR T cells and L-NIL resulted in superior tumor control (**Extended Data Fig. 8A and B**). These results demonstrate that IFN-g-producing CAR T cells stimulate iNOS in macrophages at the tumor site, and inhibition of iNOS enhances the therapeutic effectiveness of CAR T cells.

A recent clinical study reported that iNOS in pretreatment LBCL tumors is negatively associated with clinical outcomes following axi-cel treatment [51]. A source of these iNOS+ cells can come from the blood and we observed that NDR patients have increased iNOS in circulating CD11b$^{+}$CD14$^{+}$ monocytes in pre-lymphodepletion leukaphereses (**Fig. 8H**). These monocytes can traffic to tumor and differentiate into TAMs in the TME. The immune-resistant and or immune-suppressive status of the TME is likely driven by host factors such as inflammatory status and lymphoma biology. Indeed, we found that pre-lymphodepletion serum collected from DLBCL patients modulated the immunoregulatory activity of macrophages so that serum from non-responsive (NR) patients enhanced the immunoregulatory capacity of macrophages, suppressing expansion of human CD19 CAR T cells to levels comparable to imMac. In contrast, macrophages treated with serum from complete response (CR) patients resulted in CAR T cell expansion similar to unMac (**Fig. 8I**).

## DISCUSSION

Our findings demonstrate that iNOS upregulation in imMac, provoked by IFN-γ secreted from CAR T cells, impairs various aspects of CAR T cell biology, including expansion, effector function, and metabolism, all of which can reduce the therapeutic efficacy of CAR T cells. iNOS is considered an antitumor macrophage-associated marker because of its direct tumoricidal effects and upregulation with other immune-activating molecules crucial for antigen presentation and costimulation mediated by pro-inflammatory cytokines such as IFN-γ, TNF-α, and IL-1β [52]. Our study highlights IFN-γ induction of iNOS in imMac as a critical factor that impairs CAR T cell function. This finding aligns with clinical observations across various cancers where elevated iNOS expression in tumors correlated with unfavorable prognoses, highlighting the tumor-promoting potential of iNOS [53-56]. Notably, elevated expression of iNOS in pre-CAR T cell treatment TME has been linked to unfavorable outcomes in LBCL patients treated with axi-cel [51]. Our data show that unMac expresses iNOS but they do not suppress CAR T cell activity to the same extent as imMac. This disparity might be attributed to the lower extent of iNOS and NO production in unMac compared to imMac after exposure to CAR T cells. Moreover, the coexpression of various immunosuppressive markers, including ARG1, in imMac likely amplifies their inhibitory effects on CAR T cells. Thus, excessive NO production combined with coexpressed immunosuppressive proteins can shift the microenvironment toward immune suppression and tumor progression. Our experimental design mimics the tumor niche, providing insights into how CAR T cells stimulate imMac toward a suppressive phenotype in the presence of tumor-derived signals.

Mechanistically, IFN-γ induction of iNOS within imMac upregulates the p53 pathway while downregulating MYC targets in CAR T cells. Elevated levels of iNOS and NO may expose CAR T cells to oxidative and nitrosative stress, triggering damage to DNA and other cellular components. This damage activates the p53 pathway promoting cell cycle arrest, apoptosis, and DNA damage repair to prevent the propagation of damaged DNA resulting in proliferative arrest [57]. Simultaneously, the downregulation of MYC target proteins, which are critical for ribosome biogenesis, amino acid uptake, and protein translation, hinders protein synthesis in response to immune activation, thereby compromising CAR T cell responses [58].

Our study also reveals that IFN-γ dependent, iNOS-mediated CAR T cell dysfunction involves repression of glycolytic and oxidative metabolic capacity. The metabolic profiles of CAR T cells are crucial for their antitumor activity, persistence, and differentiation into memory T cells [59,60]. Notably, we observed a rewiring of the TCA cycle, leading to itaconate accumulation in CAR T cells triggered by iNOS-expressing imMac. While IRG1 expression and itaconate production have been previously identified in macrophages and MDSCs as immunosuppressive mediators, their stabilization and functional roles in T cells remain unexplored [49,61]. This metabolic rewiring likely arises from the NO-mediated disruption of iron-sulfur (Fe-S) clusters in key TCA cycle enzymes, such as aconitase and succinate dehydrogenase (complex II). Inhibition of these enzymes leads to the accumulation of citrate and succinate, both of which contribute to the stabilization of HIF1-a, triggering a hypoxia-like response [62,63]. Furthermore, NO-mediated inhibition of complexes I and IV in the mitochondrial electron transport chain (ETC) impairs oxidative phosphorylation, further exacerbating CAR T cell metabolic dysfunction and compromising their antitumor efficacy.

Our work highlights IFN-γ as a key initiator of the iNOS-dependent inhibitory circuit between CAR T cells and imMac. The role of IFN-γ in the TME and its impact on CAR T cells is pleiotropic. In the TME it can promote tumor cell apoptosis and activate cellular immunity but can also upregulate inhibitory molecules, such as PD-L1, PD-L2, indoleamine 2,3-dioxygenase 1 (IDO), FAS, and FAS ligand (FASL) [64]. During CAR T cell therapy, IFN-γ enhances host antitumor immunity and potentiates CAR T cell-mediated tumor control [40,42,65]. The efficacy of IFN-γ-driven antitumor responses depends on the intrinsic sensitivity of cancer cells to IFN-γ-induced cell death [66] and the capacity of IFN-γ receptor signaling to stabilize the immunologic synapse between CAR T cells and their targets [67]. In addition to enhancing CAR T cell function IFN-γ can induce iNOS in macrophages critical for inducing or maintaining CRS toxicity [25,41]. In another study, we determined that IFN-γ can also induce cytopenias and its blockade can rescue mice from CRS and cytopenias[68], confirming pilot studies of IFN-γ blockade in patients with severe CRS [69]. In contrast, we now report that blocking IFN-γ mitigates the suppressive effects of imMac and improves CAR T cell function. Therefore, the impact of IFN-γ on the TME and CAR T cells is likely determined by patient-dependent factors such as inflammatory status, TME, and CAR T product, as well as IFN-γ blockade factors such as dose and timing. Our work supports targeting IFN-γ to improve clinical responses to CAR T therapy but host-dependent factors should guide patient selection with information derived from transcriptomic, genomic, or inflammatory status as we have reported [16,20,70,71]. Translational efforts targeting IFN-γ may enhance the probability of achieving more frequent durable responses from CAR T cell therapy in patients with hematologic malignancies and guide clinical trials of CAR T cells for solid tumor malignancies.

## MATERIALS AND METHODS

### Patient samples

All samples were prospectively obtained from patients with relapsed or refractory LBCL who underwent axi-cel treatment at the H. Lee Moffitt Comprehensive Cancer Center or the Roswell Park Comprehensive Cancer Center. The collection of samples was conducted in accordance with approved protocols by the institutional review board. Pre-treatment tumor biopsies were obtained within 1 month prior to axi-cel infusion and before lymphodepletion. Serum samples were also collected at indicated timepoints. Patients who achieved sustained remission for at least 6 months following axi-cel infusion were classified as durable responders (DR). Non-durable responders (NDR) were patients who either experienced lymphoma relapse or passed away due to recurrent disease.

### Mice

All animal studies were performed according to a protocol approved at the Institutional Animal Care and Use Committee at the H. Lee Moffitt Cancer Center and Research Institute, the University of South

Florida, or the Roswell Park Comprehensive Cancer Center. C57BL/6J mice, *Nos2*$^{-/-}$ (B6.129P2-*Nos2*$^{tm1Lau}$/J) mice, *Ifng*$^{-/-}$ (B6.129S7-*Ifng*$^{tm1Ts}$/J) mice, and *Rag1*$^{-/-}$ mice (B6.129S7-*Rag1*$^{tm1Mom}$/J) were purchased from Jackson Laboratories. *Rag1*$^{-/-}$ mice were bred in-house.

#### Cell lines

Eμ-myc cells were derived from the axillary lymph node of tumor-bearing Eμ-myc transgenic mice, a spontaneous lymphoma model [72-74]. For some experiments, Eμ-myc cells that were retrovirally transduced to express GFP-firefly luciferase (Eμ-myc-GFP-FFL) were used. Eμ-myc cells were maintained on irradiated (30 Gy) NIH-3T3 feeder cells in RPMI-1640/IMDM (1/1, v/v) supplemented with 10% heat-inactivated fetal bovine serum (HI-FBS), 2 mM L-glutamine, 100 U/ml Penicillin/Streptomycin, and 22.5 μM b-mercaptoethanol. Prior to use as feeder cells, NIH/3T3 was maintained in DMEM supplemented with 10% HI-FBS, 2 mM L-glutamine, and 100 U/ml Penicillin/Streptomycin. Raji cells were maintained in RPMI1640, 10% FBS, 2 mM L-glutamine, 100 U/ml Penicillin/Streptomycin. Cell lines were routinely tested for the absence of mycoplasma contamination using the Universal Mycoplasma Detection kit (ATCC) or MycoAlert PLUS mycoplasma detection kit (Lonza).

#### Patient tumor bulk RNA-sequencing

RNA-sequencing was performed as described [20,70]. Formalin-fixed paraffin-embedded (FFPE) or snap-frozen samples were obtained and examined by a hematologist for tumor content. RNA was extracted and RNA-sequencing libraries were prepared using NuGen RNA-Seq Multiplex System (Tecan US) according to the manufacturer's protocols. The libraries were then sequenced on the Illumina NextSeq 500 system with a 75-base paired-end run at 80 to 100 million read pairs per sample. To determine the immune cell composition in bulk RNA-seq profiles of tumor biopsies, we applied CIBERSORTx v.1.0.41 (https://cibersortx.stanford.edu) with the LM22 signature matrix. Geneset enrichment analysis of M2-associated gene expression was performed on the R package GSVA, utilizing a panel of genes as previously described [75].

#### Patient leukapheresis sample flow cytometry analysis

Cryopreserved cells from apheresis were removed from liquid nitrogen and rapidly thawed in a 37˚C water bath prior to being transferred to 10 ml of pre-warmed complete media to remove excess DMSO. Cells were centrifugated 5 min at 1500rpm, the cell pellet was washed twice with PBS and resuspended in 100 μl of a solution containing 1X Live/Dead Fixable NIR stain (Invitrogen, ThermoFisher Scientific) and 1μL of human Fc-receptor block (BD). Cells were incubated for 30 min at room temperature. Surface staining was performed for 30 min at 4°C with antibody mix in MACS buffer with 0.5% BSA (Miltenyi Biotec). Cells were then fixed using IC Fixation Buffer (eBioscience) for 30 min at RT, washed 1X Permeabilization Buffer (eBioscience), and intracellular staining was performed for 30 min at 4°C with antibody mix in 1X Permeabilization Buffer (eBioscience). Samples were analyzed with a Symphony flow cytometer (BD Biosciences) and data analyzed using FlowJo software. The following monoclonal antibodies were obtained from BD Biosciences: anti-CD45 (HI30), anti-HLD-DR (L203.rMAb), anti-CD11b (ICRF44), anti-CD33 (WM53), anti-CD15 (HI98) and anti-CD116 (hGMCSFR-M1). Anti-ARG1 (A1exF5) was obtained from Thermo Fisher, and anti-CD14 (HCD14) and anti-iNOS (W16030C) from Biolegend. Anti-CD3 (R&D Systems), anti-CD20 (Biolegend), and anti-CD56 (Biolegend) were used to dump non-myeloid cells.

#### Generation of retroviral constructs

Plasmids encoding 19dz and 1928z CAR constructs in SFG g-retroviral vectors have been described [34]. The murine 1928z CAR construct includes anti-murine CD19 scFv (1D3), murine CD8a transmembrane and hinge domains, murine CD28 intracellular domain, and murine CD3z intracellular domain, followed by the mCherry reporter via glycine-serine linker. The murine 19dz CAR construct includes the same sequence as 1928z construct except for absence of CD28 intracellular domain and having a truncated CD3z intracellular domain. Human 1928z CAR construct includes FMC63 scFv with CD8α transmembrane and hinge domain, followed by human CD28 and CD3z intracellular domains, and a GFP reporter linked via glycine-serine linker. For retrovirus production, plasmids were transfected to H29 cell lines using a calcium phosphate transfection kit (Invitrogen) to produce vesicular stomatitis virus G-glycoprotein-pseudotyped retroviral supernatants. These retroviral supernatants were subsequently used to transduce Phoenix-ECO or RD114 cell lines, which stably produce retroviral particles pseudotyped with Moloney murine leukemia virus or feline endogenous virus, respectively.

#### Mouse T cell isolation and CAR T cell generation

Mouse spleens were excised, mechanically disrupted, and filtered through a 40 μm cell strainer.

$CD3^+$ T cells were enriched via negative selection using EasySep Mouse T Cell Isolation Kit (STEMCELL Technologies). T cells were activated and expanded with anti-CD3/28 Dynabeads (Gibco) at a bead-to-cell ratio of 0.8:1. T cells were spinoculated (2000×g, 1 h, room temperature) twice, 24 h and 48 h after initial T cell activation, with viral supernatants collected from Phoenix-ECO cells on retronectin (Takara) coated plates. Following the second spinoculation, T cells were maintained for one day. On day 5, anti-CD3/28 Dynabeads were removed, and CAR T cells were used for *in vitro* or *in vivo* experiments. CAR transduction efficiency was determined by flow cytometry as a percentage of $mCherry^+$ cells in live cells. Mouse T cells were cultured in RPMI-1640 supplemented with 10% HI-FBS, 2 mM L-glutamine, 100 U/ml Penicillin/Streptomycin, 1× nonessential amino acids, 1 mM sodium pyruvate, 10 mM HEPES, 55 μM 2-mercaptoethanol, and 100 IU/ml recombinant human IL-2.

#### Human T cell isolation and CAR T cell generation

Human peripheral blood mononuclear cells (PBMCs) were obtained from STEMCELL Technologies. T cells were enriched via negative selection using the EasySep Human T cell Isolation Kit (STEMCELL Technologies). T cells were activated and expanded with anti-CD3/28 Dynabeads (Gibco) at a bead-to-cell ratio of 0.8:1. Spinoculation was performed twice at 24 h and 48 h post activation (2000×g, 1 h, room temperature) using viral supernatants collected from RD114 cells on retronectin (Takara) coated plates. Following the second spinoculation, T cells were maintained for one day. On day 5, anti-CD3/28 Dynabeads were removed, and CAR T cells were maintained for two additional days before being used for *in vitro* experiments on day 8. CAR transduction efficiency was determined by flow cytometry, measuring the percentage of $GFP^+$ cells in live cells. Human T cell complete medium consists of RPMI1640, 10% FBS, 2 mM L-glutamine, 100 U/ml Penicillin/Streptomycin, and 100 IU/ml recombinant human IL-2.

### Animal experiment

Six- to 10-week-old *Rag1*$^{-/-}$ mice or C57BL/6 of both sexes were intraperitoneally (i.p.) injected with $3\times10^6$ Eμ-myc-GFP-FFL cells to generate tumors localized in peritoneal cavity. Tumor engraftment was verified by bioluminescence imaging one day before CAR T cell transfer. Mice were randomized to different treatment groups without differences in pre-treatment tumor load. C57BL/6 mice received 300 mg/kg cyclophosphamide intraperitoneally one day before CAR T cell transfer. Seven days after tumor cell inoculation, mice were injected i.p. with $5\times10^6$ CAR T cells in 300 μl PBS. For survival experiments, L-NIL or PBS was administered i.p. once per day at 20 mg/kg body weight starting on the same day of tumor cell injection. Experimental endpoints were achieved when mice demonstrated signs of morbidity or hind-limb paralysis, or when solid tumor masses reached 2000 $mm^3$ for some mice that developed palpable masses. Bioluminescence imaging was performed by IVIS Lumina III In Vivo Imaging System (PerkinElmer) with Living Image software (PerkinElmer).

### Murine macrophage development and polarization

BMDMs were generated from bone marrow cells harvested from femurs and tibias of WT or iNOS$^{-/-}$ mice. Following red blood cell lysis by ACK (Ammonium-Chloride-Potassium) lysis buffer, $1\times10^7$ bone marrow cells were cultured in 10-cm tissue culture dish in 10 ml of RPMI-1640 supplemented with 10% HI-FBS, 2 mM L-glutamine, 100 U/ml Penicillin/Streptomycin, and 20 ng/ml M-CSF (R&D systems) for 7 days. On day 3, 10 ml of fresh medium with 20 ng/ml M-CSF was added. On day 5, the culture medium was entirely discarded and replaced by 15 ml of fresh medium with 20 ng/ml M-CSF. On day 6, BMDMs were activated for 24 h with 20 ng/ml of IL-4 and IL-10 (Peprotech) [76,77] to develop imMac or cultured in media only to use as unMac. M-CSF (20 ng/ml) was added during activation with cytokines. On day 7, adherent cells were harvested by gentle scraping and used for *in vitro* experiments.

### Human macrophage development and polarization

Monocytes were enriched from PBMCs using the EasySep Human Monocyte Enrichment Kit (STEMCELL Technologies) and were seeded in a 96-well plate at the concentration of $2.5\times10^5$ cells/ml in 200 μl of RPMI-1640 supplemented with 10% HI-FBS, 2 mM L-glutamine, 100 U/ml Penicillin/Streptomycin, and 25 ng/ml M-CSF (R&D systems). On day 3, 100 μl of the culture medium was removed and replaced with 100 μl of fresh medium containing 25 ng/ml M-CSF. On day 5, the culture medium was entirely discarded and replaced with 150 μl of fresh medium with 25 ng/ml M-CSF. On day 7, monocyte-derived macrophages (MDMs) were treated for 24 h with 20 ng/ml of IL-4, IL-10, and IL-13 (Peprotech) to develop imMac, or were cultured in media only as unMac, or in patient serum. M-CSF (25 ng/ml) was added during activation with cytokines or patient serum. On day 8, all the supernatants were removed, and MDMs were left on original plates and briefly washed with PBS before coculturing with CAR T cells and Raji cells.

#### Mouse peritoneal cell collection

Peritoneal cells were obtained by peritoneal lavage as described [25]. After euthanizing mice, 5ml ice-cold PBS/2mM EDTA were i.p. injected. Bellies were massaged for one minute and subsequently incised to drain the lavage fluid in a collection tube. Cells were filtered through a 40 μm cell strainer. Following red blood cell lysis with ACK lysing buffer, peritoneal cells were used for analyses. For *ex vivo* coculture experiments with CAR T cells, EasySep Mouse CD11b Positive Selection Kit II (STEMCELL Technologies) was used to isolate $CD11b^+$ myeloid cells.

#### Expansion assay

The expansion of $mCherry^+$ CAR T cells was determined by an IncuCyte S3 live cell analysis system (Essen Bioscience). $2\times10^4$ Eμ-myc cells and $2\times10^4$ CAR T cells were cocultured in the absence or presence of $0.5\times10^4$ macrophages (CAR T:Eμ-myc cell:Macrophage=1:1:0.25, unless otherwise indicated in the figure legends) in a 96-well black-walled clear bottom plate in 120 μl of media. Cell images were captured at 4X magnification. The expansion index was calculated by dividing the total integrated red intensity (RCU × $\mu m^2/mm^2$) at each time point by the first time point.

The expansion of human $GFP^+$ CAR T cells was determined by an IncuCyte S3 live cell analysis system (Essen Bioscience). $2\times10^4$ Raji cells and $2\times10^4$ CAR T cells were cocultured in the presence of macrophages from the same donor in a 96-well black-walled clear bottom plate in 120 μl of media. Cell images were captured at 4X magnification. The expansion index was calculated by dividing the total integrated green intensity (GCU × $\mu m^2/mm^2$) at each time point by the first time point.

#### Griess assay

$2\times10^4$ Eμ-myc cells and $2\times10^4$ CAR T cells were cocultured in the presence or absence of $0.5\times10^4$ macrophages in a 96-well plate in 120 μl of media. Coculture supernatants were harvested, and nitric oxide levels were measured using Griess reagent system (Promega) according to manufacturer's instructions. Absorbance was read at 560 nm using microplate reader (GloMax, Promega), and $NO_2^-$ concentrations were determined by standard curve. Standard curve was prepared with diluting 0.1M sodium nitrite standard (provided in the kit) with the culture media used for experiments.

#### BrdU incorporation assay

$2{\times}10^5$ Eμ-myc cells and $2{\times}10^5$ CAR T cells were cocultured in the absence or presence of $0.5{\times}10^5$ macrophages in a 24-well plate in 1200 μl of media. At 24 h of coculture, BrdU was added to each well at 10 μM. After an additional incubation for 18 h, cells were harvested. BrdU staining was performed according to APC BrdU flow kit (BD Pharmingen) and BrdU incorporation was analyzed by flow cytometry.

#### Flow cytometry

The following fluorophore-conjugated anti-mouse antibodies were used. From BD Horizon: anti-CD45 (30-F11), anti-CD19 (1D3), anti-CD11b (M1/70), and anti-CD3e (145-2C11). From BioLegend: anti-CD8a (53-6.7), anti-PD-L1 (10F.9G2), and anti-F4/80 (BM8). From eBioscience: anti-mouse ARG-1 (A1exF5) and anti-NOS2 (CXNFT). Fc receptors were blocked using FcR Blocking Reagent (anti-mouse CD16/CD32 antibody, Invitrogen). DAPI (BD Pharmingen) and Zombie NIR Fixable Viability Kit (BioLegend) were used as viability dyes. For intracellular staining, surface-labeled cells were fixed and permeabilized with Cytofix/Cytoperm kit (BD Biosciences) according to the manufacturer's instructions and then stained with intracellular antibodies. For cell surface CAR staining, protein L-biotin conjugate followed by PE-conjugated streptavidin was used. Flow cytometry was performed on a LSR II or FACSymphony instrument (BD Biosciences). Data were analyzed with the FlowJo software (FlowJo LLC).

#### CAR T cell isolation from initial coculture for subsequent *downstream assays*

CAR T cells ($5{\times}10^5$ cells/ml) and Eμ-myc-GFP-FFL cells ($5{\times}10^5$ cells/ml) were cocultured with or without macrophages ($1.25{\times}10^5$ cells/ml) for 48 hours. The cultures were prepared in either a 6-well plate with 2 ml of per cell type (CAR T cells, Eμ-myc cells, and macrophages), totaling 6 ml, or in a 10-cm dish with 12 ml of per cell type, totaling 36 ml. After initial coculture, cells were harvested, and T cells were isolated using Mouse T Cell Isolation Kit (STEMCELL Technologies). T cell purity was 100% as tested by flow cytometry. Percentage of CAR-expressing T cells was determined with flow cytometry and were subsequently used for *downstream assays*.

#### Luciferase-based killing assay

$2{\times}10^4$ Eμ-ALL-GFP-FFL cells were cocultured with CAR T cells at different effector-to-target ratios in a 96-well white-walled plate in 100 μl of media. Following incubation, 100 μl luciferase substrate reagent (ONE-Glo Luciferase assay system, Promega) was added to each well. Target cells alone were plated at the same cell density to determine maximum luciferase signals. Emitted luminescence was detected in the microplate reader (GloMax, Promega). Percent lysis was determined as (1- sample signal/maximum signal)×100.

#### Cytokine secretion assay

$2{\times}10^4$ Eμ-myc-GFP-FFL cells were cocultured with $2{\times}10^4$ CAR T cells in a 96-well plate in a total volume of 100 μl of media. Supernatants were collected and analyzed for IFN-g and TNF-a secretion using Ella automated immunoassay system (Proteinsimple Bio-techne) according to manufacturer's instructions.

#### Immunoblotting

T cells were lysed in ELB lysis buffer (50 mM HEPES, pH 7.5, 250 mM NaCl, 5 mM EDTA, 0.5 mM DTT, 0.1% NP-40 alternative, 10 μg/ml aprotinin, 10 μg/ml leupeptin, and 100 μg/ml trypsin/chymotrypsin inhibitor). Following protein quantification with the Pierce BCA protein assay (ThermoFisher), the samples were mixed with a loading buffer containing 2-mercaptoethanol. The proteins were electrophoresed in 4-20% Tris-Glycine gels (Novex-Invitrogen) and transferred to PVDF membrane with a Bio-Rad Trans-Blot SD Semi-Dry Transfer Cell. The membrane was blocked in 5% bovine serum albumin (BSA) in TBST and subsequently blotted with primary and secondary antibodies in 5% BSA in TBST. The following antibodies were used: IDH1 (clone D2H1; Cell signaling, #8137S), IDH2 (clone D2E3B; Cell signaling, #56439S), IRG1 (clone E5B2G; Cell signaling, #19857S), β-actin (clone AC-74, Sigma-Aldrich, # A2228), and horseradish peroxidase-conjugated secondary antibodies (Donkey-anti-Rabbit, Cytiva, #NA934-1ML); Sheep-anti-Mouse, Cytiva, #NA931-1ML). Membranes were imaged with a ChemiDoc Imaging System (BioRad, #17001401) and exported through ImageLab (Bio-Rad #12012931).

#### Metabolomics and $^{13}C_6$-labeled glucose tracing analyses

For global metabolomics analysis of cell-cultured medium, the cell-free medium was obtained by performing rapid centrifugation (17,000×g, 10 sec, room temperature) to collect the supernatant. The metabolites present in 20 μl of the cell-cultured medium were then extracted using 80 μl of ice-cold MeOH. Following a 30 min incubation on ice and subsequent centrifugation (17,000×g, 20 min, 4 °C), the supernatant was subjected to LC-HRMS analysis.

Global metabolomic profiling and $^{13}C_6$-labeled glucose tracing of CAR T cells, $1\times10^6$ T cells were resuspended in either RPMI-1640 medium (RPMI + 10% heat-inactivated dialyzed FBS) or $^{13}C_6$-glucose substituted RPMI-1640 medium (glucose-free RPMI + 10% heat-inactivated dialyzed FBS + 11.1 mM $^{13}C_6$-glucose). After 4 h incubation, cells were collected, rapidly centrifuged (17,000×g, 10 sec, room temperature), and medium was removed. T cells were washed with 1 ml of ice-cold PBS, and metabolites were extracted with 300 μl of 80% methanol via incubation at -80 °C for 15 min. Samples were centrifuged (17,000×g, 20 min, 4 °C), and supernatants were transferred to an Eppendorf tube and dried in a vacuum evaporator overnight. The dried extracts were resuspended in 20 μl of aqueous 50% methanol, clarified by centrifugation (17,000×g, 20 min, room temperature), and analyzed by LC-HRMS.

LC-HRMS analysis was performed on a Vanquish UPLC coupled with a Q-Exactive HF mass spectrometer, employing the same conditions as the previously established methods [78]. A ZIC-pHILIC LC column (4.6 mm inner diameter × 150 mm length, 5 μm particle size, MilliporeSigma, Burlington, MA) with a ZIC-pHILIC guard column (4.6 mm inner diameter × 20 mm length, MilliporeSigma, Burlington, MA) was used for chromatographic separation at a column temperature of 30 °C. The mobile phases consisted of 10 mM $(NH_4)_2CO_3$ and 0.05% $NH_4OH$ in $H_2O$ for mobile phase A, and 100% can for acetonitrile (ACN) mobile phase B. The LC gradient conditions were as follows: 0 to 13 min: a decreasing of 80% to 20% of mobile phase B, 13 to 15 min: 20% of mobile phase B. The ionization was set to negative mode, with the MS scan range set to 60 to 1000 m/z. The mass resolution was 70,000, and the AGC target was 1 x $10^6$. The sample loading volume was 5 µl. The unlabeled or $^{13}C$-labeled metabolite peaks were extracted using EL-Maven with a metabolite standard-based in-house library. For global metabolomic profiling, peak areas of metabolites were normalized by the median value of the total for identified metabolite peak areas in each sample. For the $^{13}C$-labeled metabolite peaks, the natural isotope peak area was corrected using IsoCor (Version 2.2) [79].

#### Proteomics and phospho-peptide analyses

$1.5\times10^7$ T cells were lysed in denaturing lysis buffer containing 8M urea, 20 mM HEPES (pH 8), 1 mM sodium orthovanadate, 2.5 mM sodium pyrophosphate and 1 mM β-glycerophosphate. A Bradford assay was carried out to determine the protein concentration. The proteins were reduced with 4.5 mM DTT and alkylated with 10 mM iodoacetamide. Trypsin digestion was carried out at room temperature overnight, and tryptic peptides were then acidified with 1% trifluoroacetic acid (TFA) and desalted with C18 Sep-Pak cartridges according to the manufacturer's procedure. Peptide from each sample was labeled with TMTPro18plex reagent. The label incorporation was checked by LC-MS/MS and spectral counting. 95% or greater label incorporation was achieved for each channel. The 16 samples were then pooled and lyophilized.

After lyophilization, the peptides were re-dissolved in 400 micro liter of 20 mM Ammonium Formate, (pH 10.0). The high pH reversed phase separation was performed on a Xbridge 4.6 mm x 100 mm column packed with BEH C18 resin, 3.5 µm, 130Å. (Waters) The peptides were eluted as follows: 5% B (5 mM Ammonium Formate, 90% acetonitrile, pH 10.0) for 10 minutes, 5% - 15% B in 5 minutes, 15-40% B in 47 minutes, 40-100% B in 5 minutes and 100% B held for 10 minutes, followed by re-equilibration at 1% B. The flow rate was 0.6 ml/min, and 12 concatenated fractions were collected. Speedvac centrifuge was used to dry the peptides.

Following lyophilization, the peptides were re-dissolved in IMAC loading buffer containing 0.1% TFA and 85% acetonitrile. The phosphopeptides in each fraction were enriched using IMAC resin (Cell Signaling Technology.# 20432) on KingFisher robot (ThermoFisher). Briefly, the IMAC resin was washed once with loading buffer. The peptides were incubated with the IMAC resin for 30 min at room temperature, with gentle agitation. Ten microliters IMAC resin was added per sample. After incubation, the IMAC resin was washed twice with loading buffer followed by 1 wash with wash buffer (80% ACN, 0.1% TFA). The phosphopeptides were eluted with elution buffer (50% ACN, 2.5% Ammonia). The volume was reduced to 20 µl via vacuum centrifugation.

A nanoflow ultra high performance liquid chromatograph (RSLC, Dionex, Sunnyvale, CA) coupled to an electrospray bench top orbitrap mass spectrometer (Orbitrap Exploris480 with FAIMS, Thermo, San Jose, CA) was used for tandem mass spectrometry peptide sequencing experiments. The sample was first loaded onto a pre-column (2 cm x 100 μm ID packed with C18 reversed-phase resin, 5μm, 100Å) and washed for 8 minutes with aqueous 2% acetonitrile and 0.04% trifluoroacetic acid. The trapped peptides were eluted onto the analytical column, (C18, 75 μm ID x 25 cm, 2 μm, 100Å, Dionex, Sunnyvale, CA). The 120-minute gradient was programmed as: 95% solvent A (2% acetonitrile + 0.1% formic acid) for 8 minutes, solvent B (90% acetonitrile + 0.1% formic acid) from 5% to 38.5% in 90 minutes, then solvent B from 50% to 90% B in 7 minutes and held at 90% for 5 minutes, followed by solvent B from 90% to 5% in 1 minute and re-equilibrate for 10 minutes. The flow rate on analytical column was 300 nl/min. Two CV values (-45 and -65) were used with 1.5 second cycle time each for data dependent acquisition. Spray voltage was 2100v and capillary temperature was 300 °C. The resolution for MS and MS/MS scans were set at 120,000 and 45,000 respectively. Dynamic exclusion was 15 seconds for previously sampled peptide peaks.

MaxQuant [80] (version 1.6.14.0) was used to identify peptides and quantify the TMT reporter ion intensities. MaxQuant normalized and log transformed data were used for bioinformatics analyses. PCA was performed using PCAtools [81] (v2.14.0) to assess the overall integrity of the proteomics and phosphopeptide datasets and also to examine sample variability.

Differentially expressed proteins were identified using the limma [82] (v3.58.1). The analysis utilized the linear modeling framework of limma, and empirical Bayes moderation was applied to improve statistical robustness. Peptides with an adjusted p-value (FDR) $< 0.05$ and a log2 fold-change threshold were considered significant. Differential phosphopeptides were detected using a linear mixed statistical model modeling the processing run as a random intercept and the group level as a fixed effect (lme4 [83] and lmerTest [84] versions 1.1-35.2 and 3.1-3 respectively). Pathway enrichment analysis was performed using the Gene Set Enrichment Analysis (GSEA) preranked method implemented in the clusterProfiler [85] (v4.10.1) coupled with the MSigDB gene sets H, C2cp and C5cp using msigdbr (v7.5.1). The moderated t-statistic from the differential expression analysis (limma) was used to rank the related peptide genes' effect sizes for the GSEA analysis. Significantly enriched pathways are called using a predefined false discovery rate (FDR) threshold of 0.05. Bioinformatics' analyses were programmed and carried out using R version 4.3.3.

### Seahorse assay

ECAR and OCR were measured using a Seahorse Extracellular Flux Analyzer (Agilent Technologies). XF96 microplates were coated with CellTak a day before analyses. To assay glycolytic function, T cells were resuspended in glucose-free XF medium supplied with 2 mM L-glutamine and 1 mM sodium pyruvate and seeded at $2\times10^5$ cells in 180 μl per well. Following incubation in a $CO_2$-free incubator for 60 min at 37 °C for pH stabilization, ECAR was measured in response to 10 mM glucose, 1 μM oligomycin, and 50 mM 2-deoxyglucose. To assay mitochondrial function, T cells were resuspended in XF medium supplied with 2 mM L-glutamine,1 mM sodium pyruvate, and 10 mM glucose and seeded at $2\times10^5$ cells in 180 µl per well. Following incubation in a $CO_2$-free incubator for 60 min at 37 °C for pH stabilization, OCR was measured in response to 1 µM oligomycin, 1 µM FCCP, and 0.5 µM rotenone and antimycin.

**Single cell RNA-sequencing analysis**

T cells were isolated using Mouse T Cell Isolation Kit (STEMCELL Technologies) and macrophages were isolated using Mouse F4/80 positive selection kit (STEMCELL).

Single cell libraries were generated using the 10X Genomics platform with Chromium Next GEM Single Cell 3' Kit v3.1. Cell suspensions were first assessed with ViaStain AOPI using a Cellometer K2 automated cell counter (Nexcelom), to determine concentration, viability and the absence of clumps and debris that could interfere with single cell capture. Cells were then loaded into the Chromium X Controller (10X Genomics) where they are partitioned into nanoliter-scale Gel Beads-in-emulsion with a single barcode per cell. Reverse transcription was performed and the resulting cDNA was amplified. The full-length amplified cDNA was used to generate gene expression libraries by enzymatic fragmentation, end-repair, a-tailing, adapter ligation, and PCR to add Illumina compatible sequencing adapters. The resulting libraries were evaluated on D1000 screentape using a TapeStation 4200 (Agilent Technologies), and quantitated using Kapa Biosystems qPCR quantitation kit for Illumina (Roche). Final libraries were then pooled, denatured, and diluted to 300pM with 1% PhiX control library added. The resulting pool was then loaded into the appropriate NovaSeq Reagent cartridge and sequenced on a NovaSeq6000 following the manufacturer's recommended protocol (Illumina Inc.). Minium 20,000 reads per cell were generated for downstream bioinformatics analysis using cellranger-7.0.0 software.

The count matrices were generated using cellranger [86] software and used for data analyses using Seurat [87] R package. The cells with feature counts greater than 7500 or less than 1000, or with >15% mitochondrial read counts were filtered out from the analysis to remove dead cells or doublets. The normalized and scaled UMI counts were calculated using the SCTransform method and be regressed against the percent of mitochondrial gene. Dimension reductions including principal component analysis (PCA), UMAP and tSNE was performed using the highly variable genes. Data clustering were identified using the shared nearest neighbor (SNN)-based clustering on the first 30 principal components. SingleR [88] package was utilized to identify the immune cell types using ImmGen reference dataset. Pathway scores are calculated using AddModuleScore method of Seurat package.

## Declarations

**Acknowledgments:** This work was in part supported by the following – FACCA Grant (M.L.D), Moffitt Clinical Science Award (M.D.J), NIH/NCI Grant R01CA244328 (F.L.L), NIH Grant R01HL167232 (M.L.D), The Rustum Family Endowed Chair in Translational Research (M.L.D.), Leukemia and Lymphoma Society Clinical Scholar Award (F.L.L), Hawkins Family Endowed Chair (F.L.L), generous donations from the Hyer Family Foundation and the Thiel Family. This work has also been supported in part by Total Cancer Care, Tissue Core, Molecular Genomics Core, Biostatistics and Bioinformatics Core, Flow Cytometry Core,

Analytic Microscopy Core, Proteomics and Metabolomics Core, Small Animal Imaging Laboratory Core, and Advanced Analytical and Digital Pathology Core at the H. Lee Moffitt Cancer Center & Research Institute, an NCI designated Comprehensive Cancer Center (P30-CA076292).

**Funding:** This work has been supported by funds from the Roswell Park Cancer Center, Moffitt Cancer Center, and Seoul National University as well as the following grants/contracts:

National Institutes of Health grants R01HL167232-01 to M.L.D; R01-CA184185, R01-CA233512, R01-CA262121, P01-CA250984 Project no. 4, and P30-CA076292 to P.C.R; Florida Department of Health grant no. 20B04 to P.C.R; Kite Pharma/Gilead to R.F., M.D.J., F.L.L; Creative-Pioneering Researchers Program through Seoul National University and National Research Foundation of Korea (NRF-2022M3A9I2017587, NRF-2022R1C1C1003619) to Y.P.K.

**Author contributions:** Conceptualization, S.B.L., Y.P.K., P.C.R., and M.L.D.; methodology-investigation, S.B.L., Y.P.K., J.K.M., J.C.B., E.R., H.K., D.C.C., R.V.J., and K.R.; patient samples and data, M.D.J., R.F., F.L.L., and M.L.D.; funding acquisition, M.L.D., P.C.R, and Y.P.K; supervision, M.L.D. and P.C.R.; and all authors contributed to the writing of the manuscript.

**Competing interests:** M.L.D has received research funding from Novartis, Kite/Gilead, and CRISPR. M.L.D receives fees from Synthekine, Adicet, Bellicum, Capstan, Kite, and CARGO. M.L.D has stock or stock options with Adaptive Biotechnologies and Adicet. M.L.D has licensed CAR technology to CRISPR and Atara.

M.H. has consultancy, speaker's bureau and/or honoraria for Adaptive Biotechnologies, Amgen, Aptitude Health, Blueprint Oncology, Celgene, Decibio, Diaceutics, Guidepoint, Seattle Genetics, Stemline, Tegus, Janssen, BMS.

J.M.K has been funded by a sponsored research agreement with Bristol Myers Squibb unrelated to this project.

M.D.J has Consultancy/Advisory for Kite/Gilead, Myeloid Therapeutics, and Allogene. Research funding from Kite/Gilead, Loxo@Lilly and Incyte. M.D.J has received research funding from Mark Foundation, a Florida Acadamic Cancer Center Alliance (FACCA) grant, and the Bankhead-Coley Cancer Research Program.

F.L.L has financial and professional relationships with following organizations. Scientific Advisory Role/Consulting Fees: A2, Allogene, Amgen, Bluebird Bio, BMS/Celgene, Calibr, Caribou, Cellular Biomedicine Group, Cowen, Daiichi Sankyo, EcoR1, Emerging Therapy Solutions, GammaDelta Therapeutics, Gerson Lehrman Group (GLG), Iovance, Kite Pharma, Janssen, Legend Biotech, Novartis, Sana, Takeda, Wugen, Umoja, Phizer; Research Contracts or Grants to my Institution for Service: Kite Pharma (Institutional), Allogene (Institutional), CERo Therapeutics (Institutional), Novartis (Institutional), BlueBird Bio (Institutional), 2SeventyBio (Institutional), BMS (Institutional), National Cancer Institute (Locke PI), Leukemia and Lymphoma Society (Locke PI); Patents, Royalties, Other Intellectual Property:

Several patents held by the institution in my name (unlicensed) in the field of cellular immunotherapy.; Education or Editorial Activity: Aptitude Health, ASH, BioPharma Communications CARE Education, Clinical Care Options Oncology, Imedex, Society for Immunotherapy of Cancer.

**Data and materials availability:** RNA-seq data have been deposited to the Gene Expression Omnibus database (accession number GSE153439).

## Figures

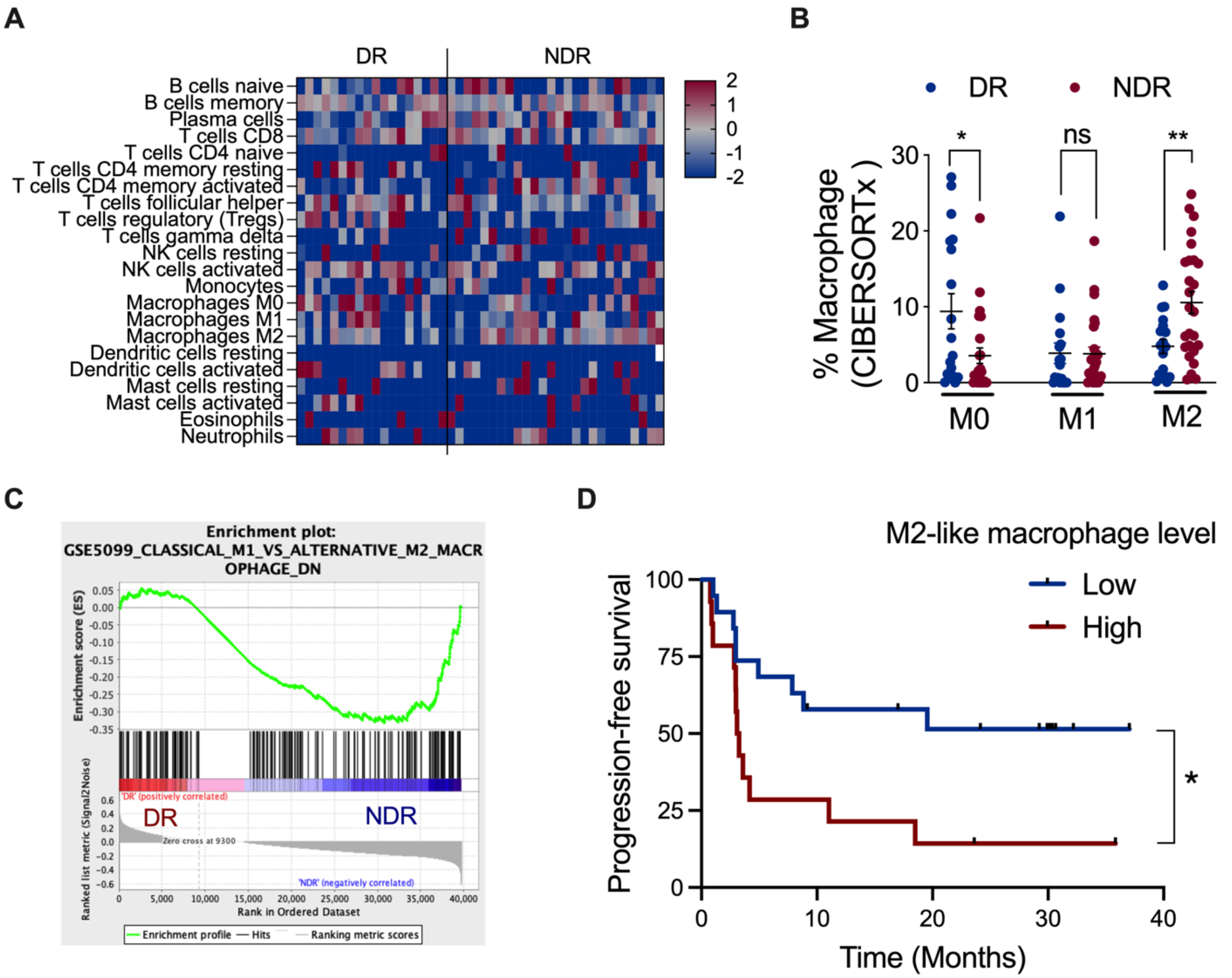

### Figure 1

**Macrophages in the pre-CAR T cell treatment TME are linked to therapeutic responses to CAR T cell therapy in LBCL patients.** Bulk RNA-seq analysis on patient tumor biopsies taken before lymphodepletion conditioning therapy and axi-cel infusion (DR, 18 patients; NDR, 26 patients). (**A**) Heatmap showing relative abundances of CIBERSORTx deconvoluted immune cell types. (**B**) Percentages of M0, M1, and M2-like macrophages based on CIBERSORTx. (**C**) Gene set enrichment analysis of M2-like macrophage signatures. (**D**) Progression-free survival in patients stratified according to the abundance of CIBERSORTx-defined M2-like macrophages ("Low" represents patients with a < 5% M2 macrophage population, 14 patients; "High" represents patients with a > 10% M2 macrophage population, 19 patients). Data in (B) are the mean ± SEM. Statistical significance was determined by unpaired two-tailed *t* tests with Welch's correction (B) or log-rank Mantel-Cox test (D). *, P < 0.05; **, P < 0.01; ns, not significant; DR, durable response; NDR, non-durable response.

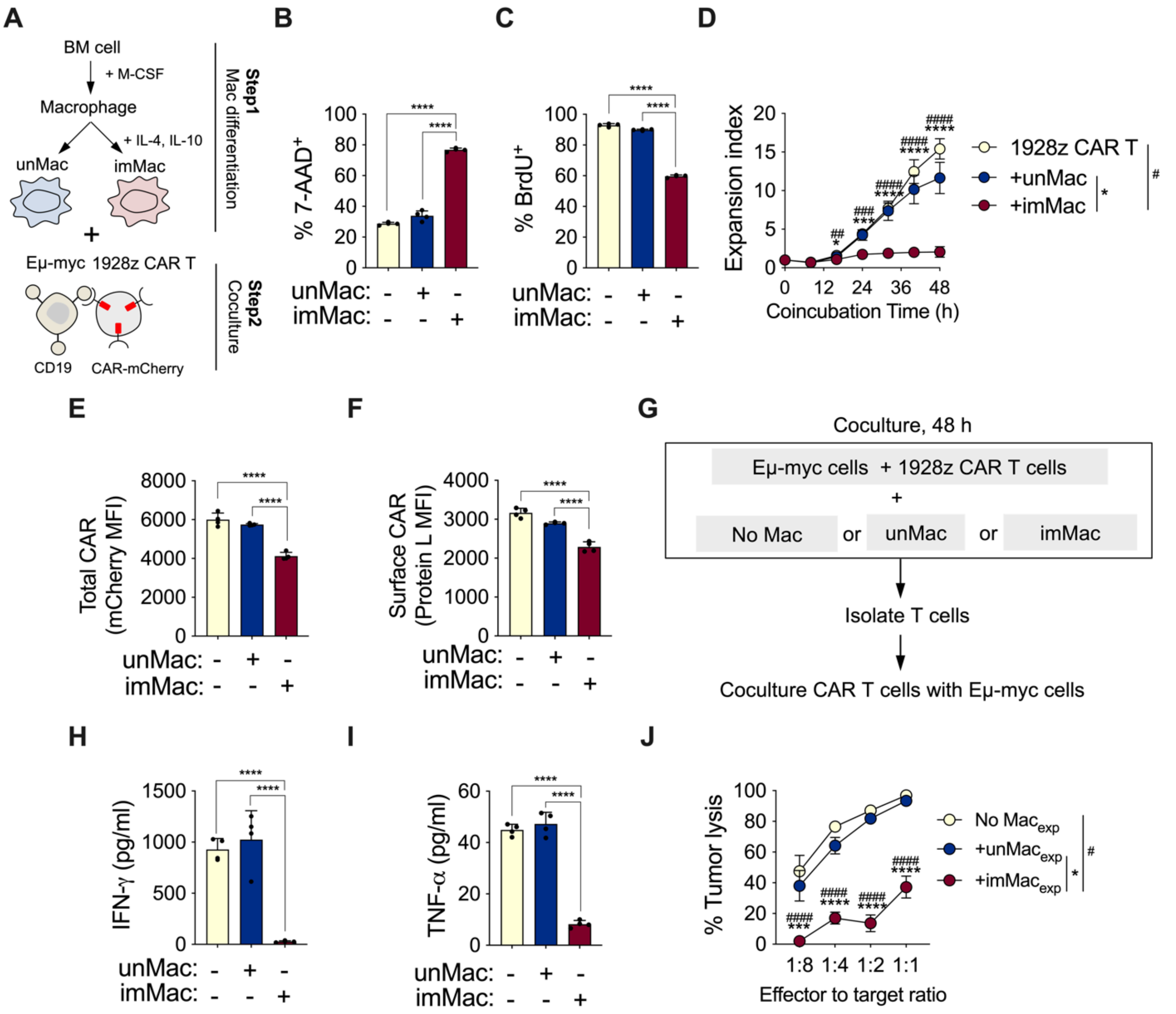

### Figure 2

**Exposure of imMac provokes CAR T cell dysfunction.** (**A**) Schematic for (B-F) where CAR T cells and Eμ-myc cells were cocultured with unMac or imMac or without macrophages. (**B**) CAR T cell death at 48 h was assessed by 7-aminoactinomycin D (7-AAD) incorporation via flow cytometry (n=4). Data are representative of n = 2 independent experiments. (**C**) DNA replication of CAR T cells at 42 h was measured by bromodeoxyuridine (BrdU) incorporation via flow cytometry (n=4). (**D**) CAR T cell expansion was measured over 48 h by a live cell analysis system (n=4). Data are representative of at least n = 3 independent experiments. (**E**) Total CAR expression levels in CAR T cells at 48 h were assessed via flow cytometry (n=4). Data are representative of at least n = 3 independent experiments. (**F**) Surface CAR expression levels in CAR T cells at 48 h were detected by staining single-chain variable fragments (scFv) of CAR with protein L and analyzed via flow cytometry (n=4). (**G**) Schematic for (H-J) where CAR T cells were isolated after initial coculture with Eμ-myc cells and with unMac or imMac or without macrophages (No Mac) for 48 h, subsequently cocultured with fresh Eμ-myc cells. (**H** and **I**) Levels of IFN-g and TNF-a in coculture supernatants of CAR T cells and Eμ-myc cells at 36 h were analyzed by an automated enzyme-linked immunosorbent assay (ELISA) (n=4). Data are representative of n = 2 independent experiments. (**J**) Lysis of Eμ-myc cells by CAR T cells at 36 h was assessed using a bioluminescence assay (n=4). Data are representative of n = 2 independent experiments. All data are the mean ± SD. Statistical significance was determined by one-way ANOVA with Bonferroni correction for multiple comparisons. *, P < 0.05; ***, P < 0.001; ****, P < 0.0001; $^{\#\#}$, P < 0.01; $^{\#\#\#}$, P < 0.001; $^{\#\#\#\#}$, P < 0.0001; BM, bone marrow; MFI, mean fluorescence intensity; exp: exposed, isolated CAR T cells from prior coculture.

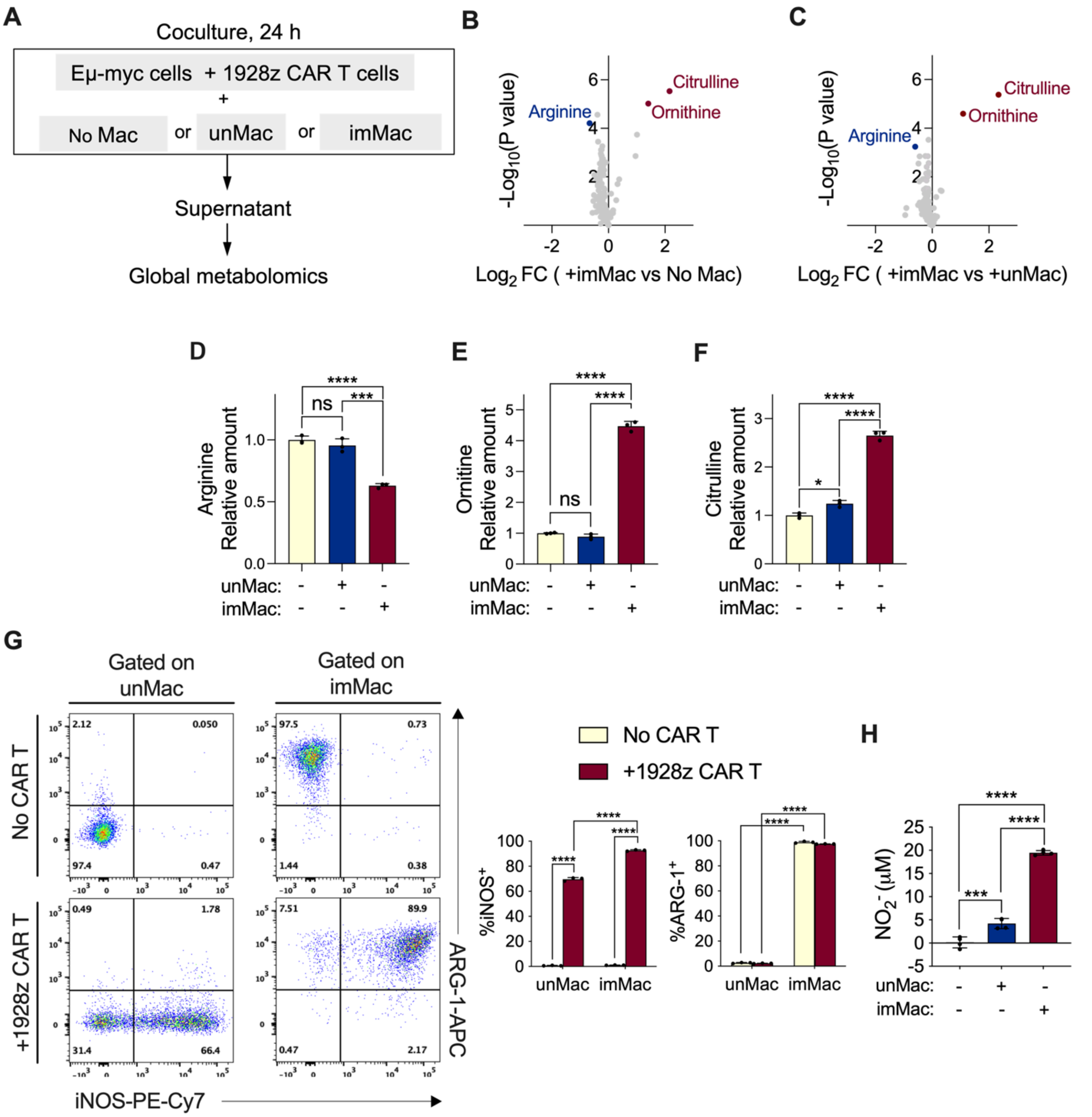

**Figure 3**

**CAR T cell-exposed imMac upregulates iNOS.** (**A**) Schematic for (B-F) where CAR T cells and Eμ-myc cells were cocultured with unMac or imMac or without macrophages (No Mac) for 24 h. Coculture supernatants were analyzed by global metabolomics using LC-MS. (**B** and **C**) Quantification of relative abundance of metabolites in the supernatants derived from cocultures containing imMac versus No Mac (B) or unMac (C). (**D**-**F**) Arginine, ornithine, and citrulline levels in the supernatants of coculture groups

(n=3). The data were normalized to the mean value of CAR T and Eμ-myc cell cocultures without macrophages. (**G**) Flow cytometry analysis of expression of ARG-1 and iNOS in unMac or imMac cocultured with Eμ-myc cells in the presence or absence of CAR T cells at 24 h (n=3). (**H**) NO levels in the supernatants derived from cocultures of CAR T cells and Eμ-myc cells with unMac or imMac or without macrophages at 48 h were analyzed by Griess assay (n=4). Data are representative of n = 2 independent experiments. Data in (D-H) are presented in mean ± SD. Statistical significance was determined by unpaired two-tailed Student's *t* tests (B, C, G) or one-way ANOVA with Bonferroni correction for multiple comparisons (D-F, H). ***, $P < 0.001$; ****, $P < 0.0001$; ns, not significant; FC, fold change; LC-MS, liquid chromatography-mass spectrometry; NO, nitric oxide.

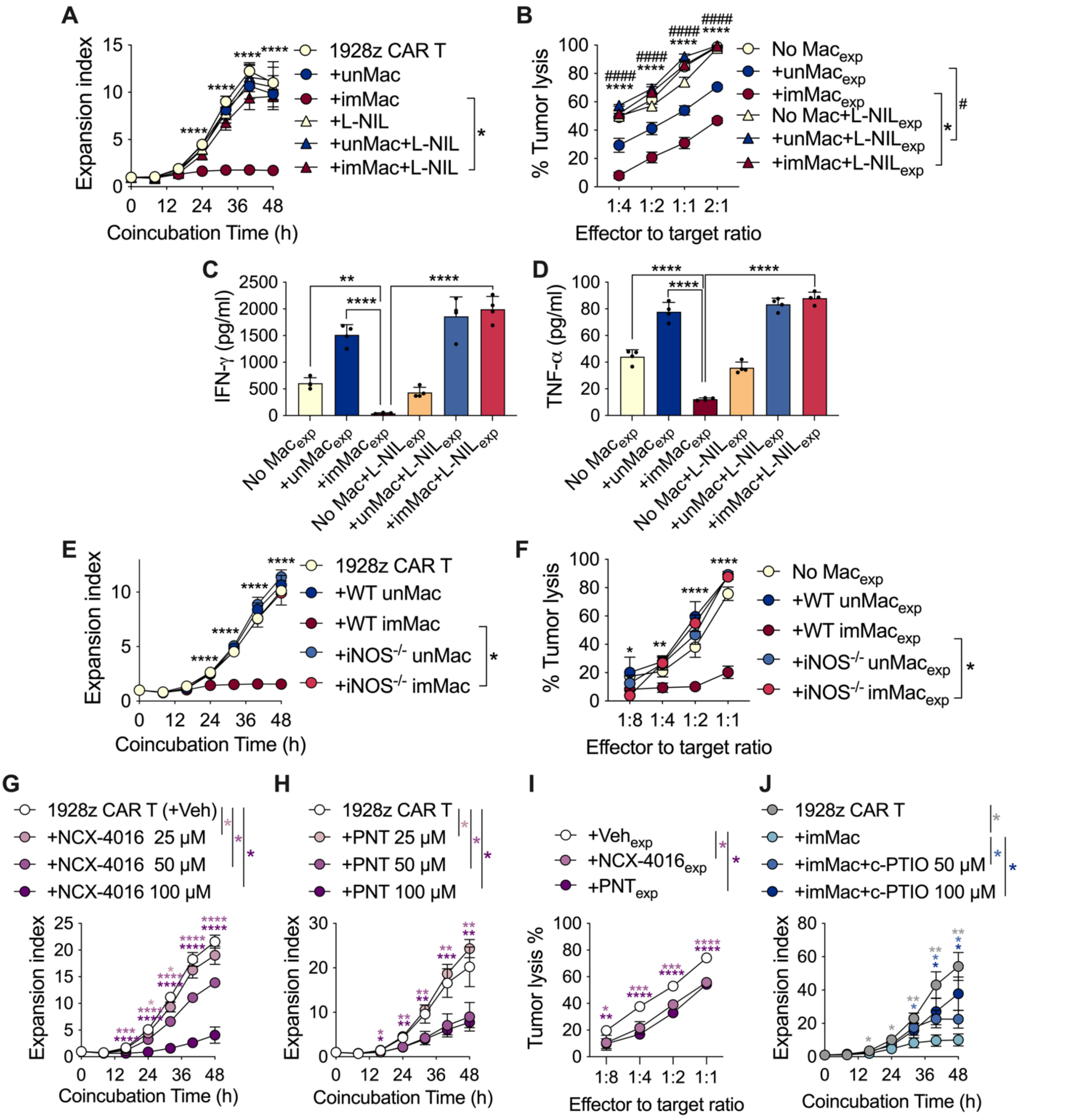

### Figure 4

**iNOS upregulation in imMac drives suppression of CAR T cell function.** (**A**) CAR T cells and Eμ-myc cells were cocultured with unMac or imMac or without macrophages in the presence or absence of L-NIL (50 mM). CAR T cell expansion was measured over 48 h by a live cell analysis system (n=4). (**B**-**D**) After initial coculture of CAR T cells and Eμ-myc cells with unMac or imMac or without macrophages (No Mac) in the presence or absence of L-NIL (50 mM) for 48 h, isolated CAR T cells were cocultured with fresh Eμ-

myc cells. (B) Luciferase-based lysis of Eμ-myc cells by CAR T cells was assessed at 24 h (n=4). Data are representative of n = 2 independent experiments. (C and D) Levels of IFN-g and TNF-a in coculture supernatants at 36 h were analyzed by ELISA (n=4). (**E**) CAR T cells and Eμ-myc cells were cocultured with WT or $iNOS^{-/-}$ unMac or imMac or without macrophages. CAR T cell expansion was measured over 48 h by a live cell analysis system (n=4). (**F**) After initial coculture of CAR T cells and Eμ-myc cells with WT or $iNOS^{-/-}$ unMac or imMac or without macrophages (No Mac) for 48 h, isolated CAR T cells were cocultured with fresh Eμ-myc cells. Luciferase-based lysis of Eμ-myc cells by CAR T cells was assessed at 36 h (n=4). (**G**) CAR T cells were cocultured with Eμ-myc cells in the presence of NCX-4016 or 0.01% DMSO (Veh). CAR T cell expansion was measured over 48 h by a live cell analysis system (n=3-4). Data are representative of n = 2 independent experiments. (**H**) CAR T cells were cocultured with Eμ-myc cells in the presence or absence of PNT. CAR T cell expansion was measured over 48 h by a live cell analysis system (n=4). Data are representative of n = 2 independent experiments. (**I**) After initial coculture of CAR T cells and Eμ-myc cells in the presence of NCX-4016 (100 mM) or PNT (50 mM) or 0.01% DMSO (Veh) for 48 h, isolated CAR T cells were cocultured with fresh Eμ-myc cells. Luciferase-based lysis of Eμ-myc cells by CAR T cells was assessed at 24 h (n=4). (**J**) CAR T cells and Eμ-myc cells were cocultured with or without imMac in the presence or absence of c-PTIO. CAR T cell expansion was measured over 48 h by a live cell analysis system (n=3). Data are representative of n = 2 independent experiments. All data are presented in mean ± SD. Statistical significance was determined by unpaired two-tailed Student's *t* tests (A, B, and E-J) or one-way ANOVA with Bonferroni correction for multiple comparisons (C, D). *, P < 0.05; **, P < 0.01; ***, P < 0.001; ****, P < 0.0001; $^{####}$, P < 0.0001; PNT, peroxynitrite; c-PTIO, carboxyl-PTIO; exp: exposed, isolated CAR T cells from prior coculture.

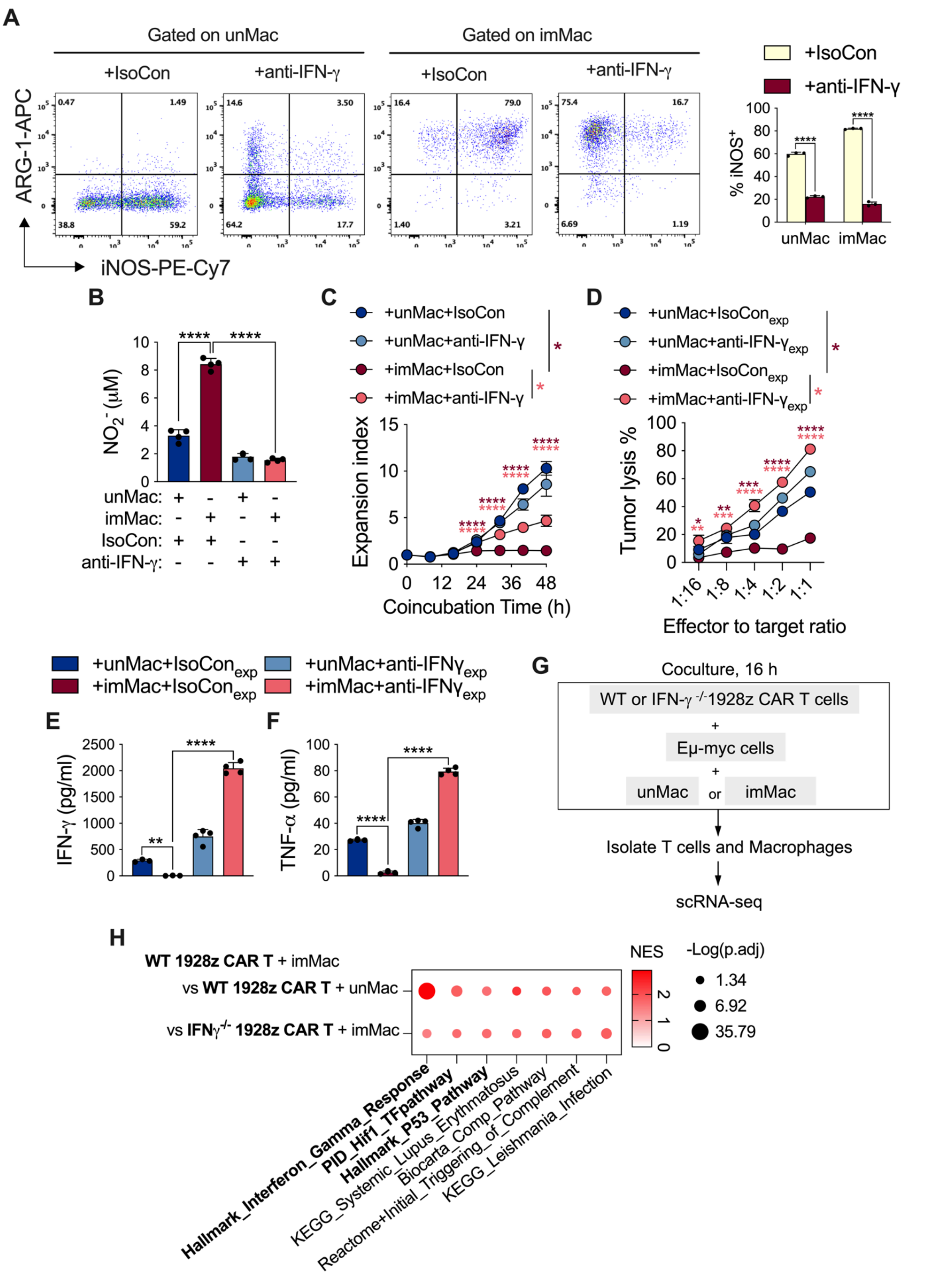

### Figure 5

**CAR T cell-derived IFN-γ induces iNOS expression in imMac. (A-C)** CAR T cells and Eμ-myc cells were cocultured with unMac or imMac in the presence of anti-IFN-g (10 mg/mL) or IsoCon (10 mg/mL). (A) Expression of ARG-1 and iNOS in unMac or imMac was analyzed at 24 h via flow cytometry (n=3). (B) NO levels in coculture supernatants at 44 h were analyzed by Griess assay (n=4). (C) CAR T cell expansion was measured over 48 h by a live cell analysis system (n=4). Data are representative of n = 2

independent experiments. (**D**-**F**) After initial coculture of CAR T cells and Eμ-myc cells with unMac or imMac in the presence of anti-IFN-g (10 mg/mL) or IsoCon (10 mg/mL) for 48 h, isolated CAR T cells were cocultured with fresh Eμ-myc cells. (D) Luciferase-based lysis of Eμ-myc cells by CAR T cells was assessed at 24 h (n=4). (E and F) Levels of IFN-g and TNF-a in the coculture supernatants at 24 h were analyzed by ELISA (n=4). (**G**) Schematic for (H) where WT or IFN-$g^{-/-}$ CAR T cells were cocultured with Eμ-myc cells in the presence of unMac or imMac. After coculture for 16 h, scRNA-seq was performed on T cells. (**H**) Pathway enrichment analysis of differentially expressed genes in CAR T cells from cocultures of WT CAR T cells and imMac, compared to those from WT CAR T cells and unMac or IFN-$g^{-/-}$ CAR T cells and imMac cocultures. Data in (A-F) are presented in mean ± SD. Statistical significance was determined by unpaired two-tailed Student's *t* tests (A, C, D) or one-way ANOVA with Bonferroni correction for multiple comparisons (B, E, F). *, P < 0.05; **, P < 0.01; ***, P < 0.001; ****, P < 0.0001; IsoCon, isotype control; NO, nitric oxide; exp: exposed, isolated CAR T cells from prior coculture.

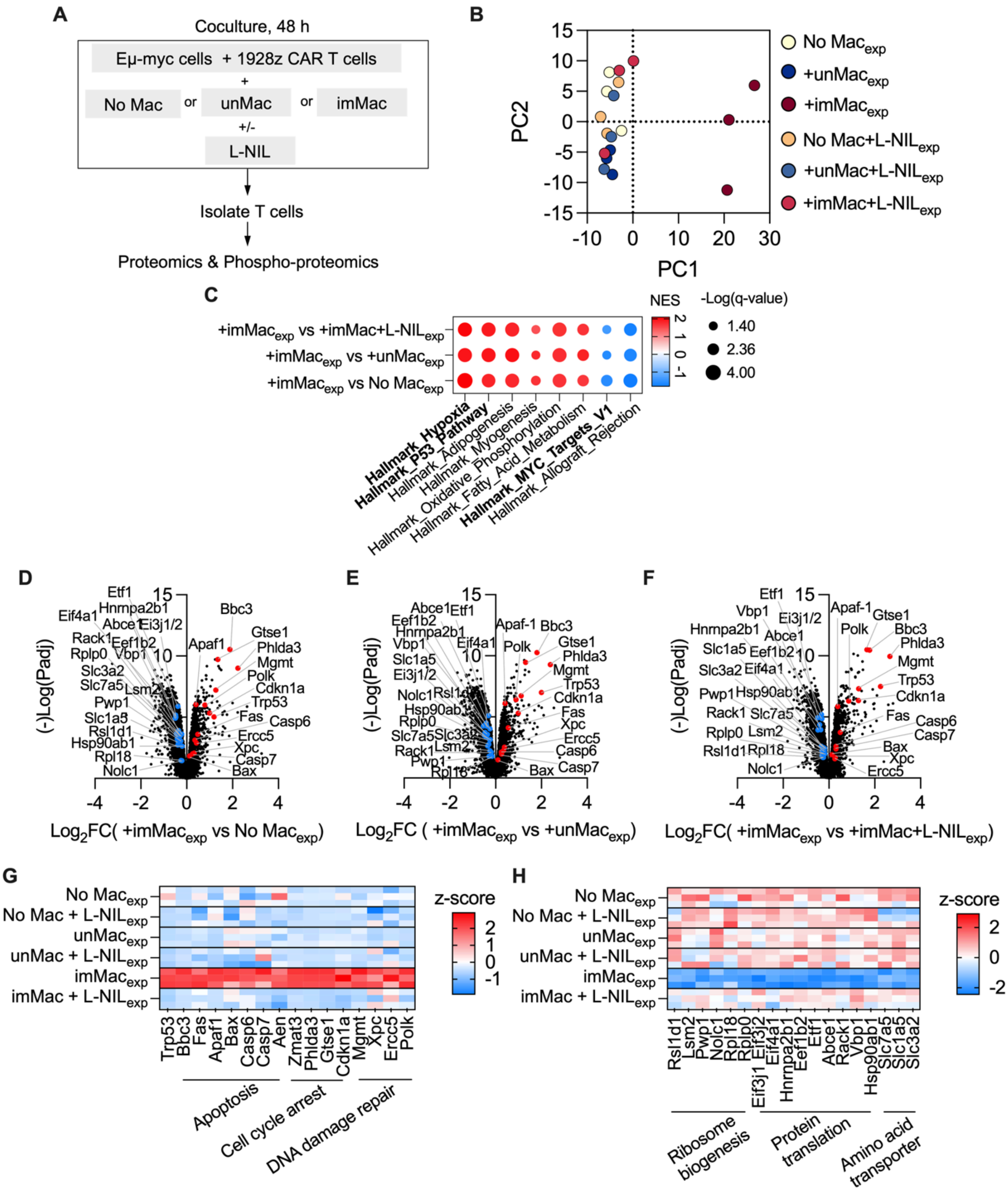

### Figure 6

**iNOS expressing-imMac drives CAR T cell dysregulation.** CAR T cells and Eμ-myc cells were cocultured with unMac, imMac or without macrophages (No Mac) in the presence or absence of L-NIL (50 mM). After coculture for 48 h, proteomics was performed on T cells. (**A**) Experimental design. (**B**) Principal component analysis (PCA) plot of proteomics data showing distinct clustering of samples (n=3). (**C**) Pathway enrichment analysis of differentially expressed proteins (DEPs) in CAR T cells cocultured with imMac versus No Mac, unMac, or imMac with L-NIL treatment. (**D-F**) The p53 and MYC pathway DEPs in CAR T cells cocultured with imMac versus No Mac (D), unMac (E), or imMac with L-NIL treatment (F). (**G**) DEPs in the p53 pathway. (**H**) DEPs in MYC target proteins.

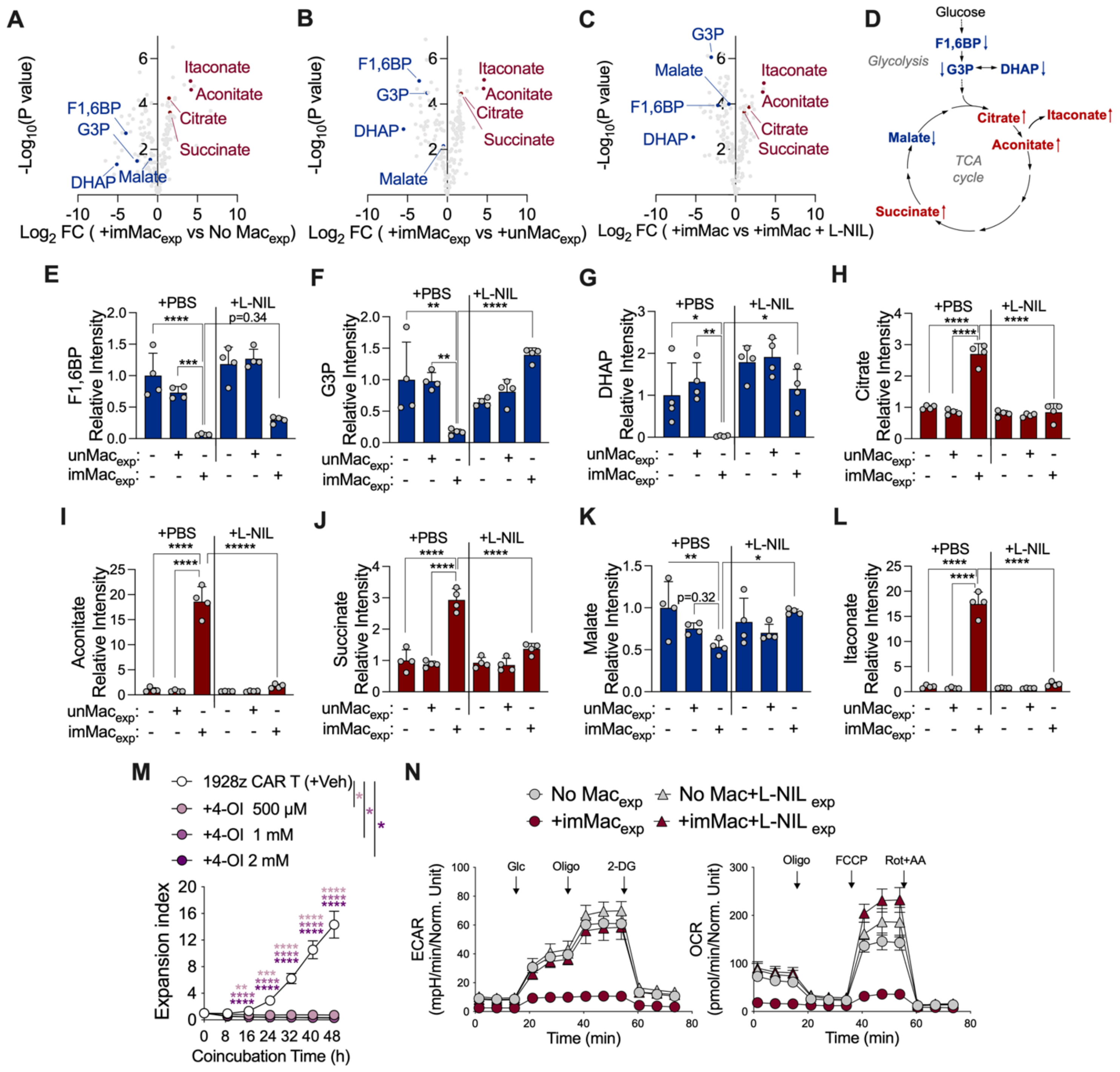

### Figure 7

**iNOS-expressing imMac induces CAR T cell metabolic dysregulation.** (**A**-**L**) CAR T cells and Eμ-myc cells were cocultured with unMac or imMac or without macrophages (No Mac) in the presence or absence of L-NIL (50 mM). After coculture for 48 h, global metabolomics was performed on T cells. Quantification of relative abundance of metabolites in CAR T cells derived from cocultures with imMac versus No Mac (A), unMac (B), or imMac with L-NIL treatment (C). (D) Schematic depicting altered metabolites associated with glycolysis pathway and TCA cycle. (E-L) Levels of F1,6BP, G3P, DHAP, citrate, aconitate, succinate, malate, and itaconate (n=4). The data were normalized to the mean value of CAR T cells derived from cocultures without macrophages and L-NIL treatment. (**M**) CAR T cells were cocultured with Eμ-myc cells in the presence or absence of 4-OI. CAR T cell expansion was measured over 48 h by a live cell analysis system (n=3-4). (**N**) CAR T cells and Eμ-myc cells were cocultured with imMac or without macrophages (No Mac) in the presence or absence of L-NIL (50 mM). After coculture for 48 h, seahorse assay was performed on CAR T cells (n=6). ECAR was measured in response to glucose (Glc), ATP synthase inhibitor (Oligo), or hexokinase II inhibitor (2-DG). OCR was measured in response to Oligo, mitochondrial oxidative phosphorylation uncoupler (FCCP), or electron transport chain complex I/III inhibitor (Rot/AA). Data in (E-L) are presented in mean ± SD. Statistical significance was determined by unpaired two-tailed Student's *t* tests (A-C, M) or one-way ANOVA with Bonferroni correction for multiple comparisons (E-L). *, P < 0.05; **, P < 0.01; ***, P < 0.001; ****, P < 0.0001; ns, not significant; F1,6BP, 1,6-bisphosphate; G3P, glyceraldehyde 3-phosphate; DHAP, dihydroxyacetone phosphate; 4-OI, 4-octyl itaconate; ECAR, extracellular acidification rate; OCR, oxygen consumption rate; Oligo, oligomycin A; 2-DG, 2-deoxy-D-glucose; FCCP, carbonyl cyanide p-trifluoromethoxyphenylhydrazone; Rot/AA, rotenone, antimycin A; exp: exposed, isolated CAR T cells from prior coculture.

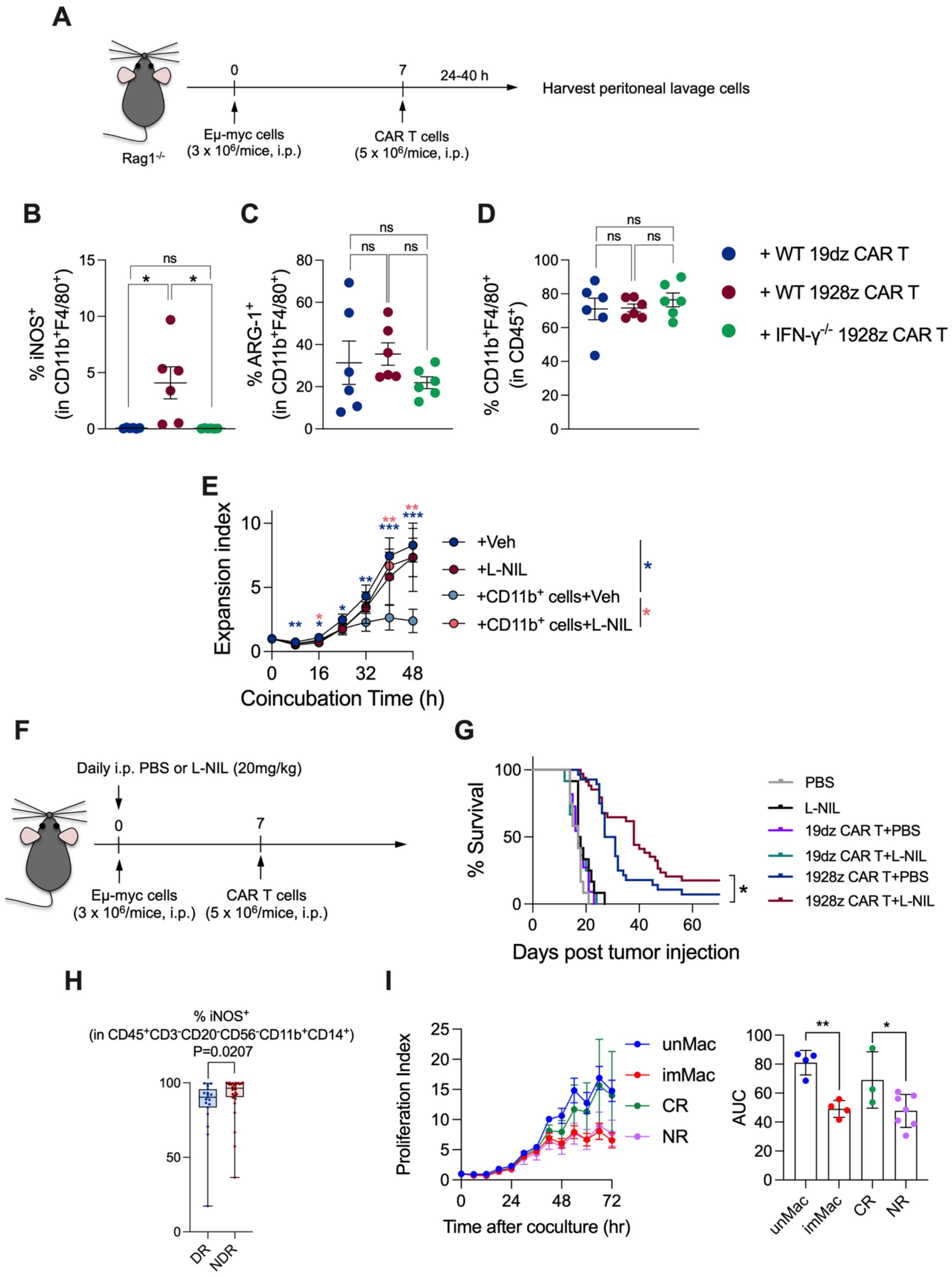

### Figure 8

**iNOS inhibition improves efficacy of CAR T cell therapy.** (**A**) Experimental settings for (B-E) where Eμ-myc cells were intraperitoneally injected into *Rag1$^{-/-}$* mice. Seven days later, WT 1928z, IFN-g$^{-/-}$ 1928z, or WT 19dz CAR T cells were transferred into mice. Peritoneal lavage cells were obtained 24-40 h after CAR T cell transfer. (**B**-**D**) Flow cytometry analysis of iNOS$^{+}$ cells among CD11b$^{+}$F4/80$^{+}$ macrophages (B), ARG-

1$^{+}$ cells among CD11b$^{+}$F4/80$^{+}$ macrophages (C), and CD11b$^{+}$F4/80$^{+}$ macrophages among CD45$^{+}$ cells (D) at 24 h after CAR T cell transfer (n=6/group). (**E**) CD11b$^{+}$ peritoneal myeloid cells were obtained 40 h following WT 1928z CAR T cell transfer (n=5 mice). CD11b$^{+}$ cells were then *ex vivo* cocultured with fresh antigen-naïve CAR T cells and Eμ-myc cells in the presence or absence of L-NIL (50 mM) (coculture ratio, 1:1:1=CD11b$^{+}$ cell:CAR T:Eμ-myc cell). Expansion of CAR T cells was measured over 48 h by a live cell analysis system (n=4-5). (**F**) Experimental settings for (G). **(G)** Percentage of survival of tumor-bearing mice treated with WT 19dz or WT 1928z CAR T cells receiving L-NIL or PBS (vehicle). Results are from two pooled independent experiments (PBS, n=12 mice; L-NIL, n=12 mice; 19dz CAR T+PBS, n=11 mice; 19dz CAR T+L-NIL, n=12 mice; 1928z+PBS, n=28 mice; 1928z+L-NIL, n=34 mice). (**H**) Percentage of iNOS$^{+}$ cells among CD45$^{+}$CD3$^{-}$CD20$^{-}$CD56$^{-}$CD11b$^{+}$CD14$^{+}$ monocytes in pre-lymphodepletion leukapheresis (DR, 19 patients; NDR, 32 patients). (**I**) Human CAR T cells were cocultured with Raji cells in the presence of human monocyte-derived unpolarized macrophages (unMac), IL-4, IL-10, IL-13-activated macrophages (imMac) or macrophages activated with serums from patients in a complete remission (CR) at 3 months post-treatment (3 patients) or non-responders (NR, 7 patients). CAR T cell expansion was measured over 48 h by a live cell analysis system. Area under the curve (AUC) values were compared. Data in (B, C, D) are presented in mean ± SEM and (E, J) are presented in mean ± SD. Statistical significance was determined by one-way ANOVA with Bonferroni correction for multiple comparisons (B, C, D), unpaired two-tailed Student's *t* tests (E, J), log-rank Mantel-Cox test (G), or two-tailed Mann-Whitney *U* test (I). *, $P < 0.05$; **, $P < 0.01$; ***, $P < 0.001$.

### Supplementary Files

This is a list of supplementary files associated with this preprint. Click to download.

- Extendeddatafigures.docx
